# Evaluation of Glyoxal fixation for immunohistochemistry of the retina

**DOI:** 10.1038/s41598-025-04226-7

**Published:** 2025-07-01

**Authors:** Glyn Chidlow, John P.M. Wood, Weng Onn Chan, Robert J. Casson

**Affiliations:** Ophthalmic Research Laboratories, Discipline of Ophthalmology and Visual Sciences, University of Adelaide, Level 7 Adelaide Health and Medical Sciences Building, North Terrace, Adelaide, SA 5000 Australia

**Keywords:** Immunohistochemistry, Retina, Fixation, Glyoxal, Formaldehyde, Retina, Immunohistochemistry

## Abstract

Immunohistochemistry has become an essential tool in retinal research. Formaldehyde is the gold standard fixative, but the development of an improved fixative is of keen interest. Herein, we performed a comprehensive evaluation of the compatibility of glyoxal fixation with retinal immunohistochemistry, using wholemounts, cryosections and paraffin-embedded eyes. Immunohistochemistry was performed in normal rat eyes, and, to facilitate localisation of stress-response proteins, eyes subjected to laser-induced retinal injury or injected with the endotoxin lipopolysaccharide. Regarding wholemounts, glyoxal fixation produced retinas that were too fragile to be consistently dissected as pristine wholemounts. In terms of antigenicity, we observed no consistent improvement when glyoxal fixation was used. Some antibodies produced higher signal intensities, but a greater number displayed weaker signal-to-background patterns of labelling compared to formaldehyde fixation. For cryosections and paraffin sections, we likewise found no compelling evidence that immunohistochemical signals were intensified when formaldehyde was replaced by glyoxal. For the 50 antibodies tested, formaldehyde typically produced signal-to-background immunolabelling that was equivalent or superior to glyoxal. In conclusion, the results of this study do not support the use of glyoxal fixation for immunohistochemistry of the rat retina, but with the caveat that improved formulations and protocols may address the limitations exposed herein.

**Supplementary Information:**

The online version contains supplementary material available at 10.1038/s41598-025-04226-7.

## Introduction

Immunohistochemistry has become an essential tool for evaluating retinal neuropathology and neurodegeneration. Routine histopathology remains valuable for determining the structural integrity of the retina and identifying any accompanying gross inflammation, but pathological changes without overt degeneration are often difficult to detect. Moreover, very limited information is imparted on disease pathogenesis. Used judiciously, immunohistochemistry can provide spatially precise, cell type-specific insights into biochemical and molecular events that occur during neurodegeneration. Furthermore, immunohistochemical assessment has become the mainstay for quantifying survival of specific neuronal populations, such as retinal ganglion cells (RGCs)^1^, in animal models of retinopathies.

Whilst immunohistochemistry is increasingly employed in retinal neuroscience, it can present a significant challenge methodologically. In order to reliably localize protein targets of diverse abundancies that are found in differing cellular compartments, the protocol used must be robust, yet sensitive to the requirements of individual antigens. Antibody validation is, of course, essential and this is a topic that has received much attention in the literature owing to the prevalence of commercial primary antibodies that have undergone only cursory testing^2,3^. Notwithstanding antibody selection, arguably the most crucial methodological determinant for achieving successful immunohistochemistry is fixation. In common with most tissues, formaldehyde-based fixation – typically comprising 4% paraformaldehyde (PFA) or 10% neutral buffered formalin (NBF) – is the customary approach employed in retinal research. Formaldehyde is a crosslinking, non-coagulating fixative. Its chief strength lies in its compatibility with the majority of proteins; sub-optimal tissue morphology, artefactual detachment of the retina, epitope masking, and compromised antibody penetration represent issues of concern^4^. Other fixatives have been employed as alternatives to formaldehyde for retinal research, including glutaraldehyde, which causes extensive cross-linking and produces excellent morphology but typically causes a pronounced loss of antigenicity^4^, and Davidson’s solution, which combines fast-acting coagulation with secondary crosslinking activity. Davidson’s fixative provides superior histological preservation to formaldehyde, while also circumventing retinal detachment, and is compatible with a wide range of antibodies^4–6^. Epitope masking with Davidson’s solution, while less evident than with glutaraldehyde, is, however, more prevalent than formaldehyde. Dehydrating coagulant fixatives, including methanol, acetone, and methanol-acetone, have also been used in retinal research and generally preserve epitopes better than cross-linking fixatives, but they suffer from cellular shrinkage and poor morphological detail. The development of an improved fixative for retinal immunohistochemistry that is compatible with paraffin- and cryo-sections as well as retinal wholemounts remains of keen interest.

Glyoxal, a small dialdehyde first tested for its utility in tissue histology over 60 years ago^7^, is the active ingredient in most commercially available, formaldehyde-substitute fixatives^8^. It is surprising, therefore, that only a handful of studies have tested its compatibility with immunolabelling. Dapson and colleagues^9,10^ utilised a commercial formulation of glyoxal and reported that it afforded rapid, uniform fixation of tissues, produced excellent morphological detail and was superior to NBF for immunohistochemistry, with many antibodies not requiring antigen retrieval. Using cell cultures and an optimized formulation of glyoxal, Richter et al.^11^ likewise demonstrated that the fixative was preferable to PFA, both for preservation of cellular morphology and for immunocytochemistry. In support of these studies, glyoxal fixation has recently been shown to be superior to PFA for immunolabelling of brain vasculature and tight junction proteins^12^, and, for immunolabelling of a wide range of ionotropic receptors, ion channels and scaffold proteins in thick cryosections as well as thin paraffin-embedded sections of brain^13^. Given these encouraging reports, the goal of the current study was to perform a comprehensive evaluation of the compatibility of glyoxal fixation with immunohistochemistry of the retina. Key aspects of the study included the use of a standardised protocol and previously validated antibodies, whether fixation in glyoxal provides superior results to formaldehyde or Davidson’s solution, and importantly, whether glyoxal is equally compatible with wholemounts, cryosections and paraffin-embedded eyes, all of which play important roles in retinal neuropathological research.

## Materials and methods

### Animals and procedures

This study was approved by the Animal Ethics Committee, University of Adelaide (Adelaide, Australia; projects M-2019-073, M-2022-061) and conformed with the Australian Code of Practice for the Care and Use of Animals for Scientific Purposes, 2013, and with the ARVO Statement for the use of animals in vision and ophthalmic research. All experiments were performed in accordance with relevant guidelines and regulations. The study is reported in accordance with ARRIVE guidelines. Adult Sprague-Dawley rats and Brown Norway rats, 8 to 12 weeks old, (Laboratory Animal Services, University of Adelaide) were housed in a temperature- and humidity-controlled room with a 12 h light/dark cycle and were provided with food and water ad libitum. Ambient lighting was maintained at < 50 lx to avoid phototoxicity. Except for those rats that received endotoxin-induced retinal inflammation or laser-induced photocoagulation burns (see below), all rats used in this study were surplus to requirements at Laboratory Animal Services, University of Adelaide and were humanely killed by exposure to a rising concentration of carbon dioxide.

For endotoxin-induced retinal inflammation, a total of 5 rats were anaesthetised with isofluorane and an intravitreal injection of 0.2% (w/v) lipopolysaccharide (LPS; 5 µl in sterile saline) was performed in 9 eyes. Rats were killed after 6 h.

Induction of retinal laser burns was carried out as previously described^14^. In brief, a total of 5 rats were anaesthetised with an intraperitoneal injection of 100 mg/kg ketamine and 10 mg/kg xylazine. The pupils were then dilated, allowing visualisation of the optimum area of retina. Using a 5.4 mm fundus laser contact lens to focus the light beam onto the retina, a frequency-doubled Nd: YAG laser with 532 nm wavelength (400 μm diameter spot size, 100 ms exposure duration, 90 mW power) was employed to produce light laser burns, defined as blanching of the fundus pigmentation and devoid of adjacent edema, subretinal or retinal hemorrhage. Both eyes of each rat received approximately 8–10 laser spots in locations around the optic nerve head. Rats were killed after 3 days.

Rats that were to be used for comparison of perfusion fixation versus immersion fixation (*n* = 10) received terminal anaesthesia (100 mg/kg ketamine plus 10 mg/kg xylazine) and were humanely euthanised by transcardial perfusion with physiological saline. For half of these rats (*n* = 5), this was followed by transcardial perfusion with glyoxal. The other half (*n* = 5) did not receive transcardial perfusion with glyoxal.

## Fixation

All eyes in the current study were enucleated within 15 min of death and immediately immersion fixed. Eyes were randomly assigned to fixative group. As mentioned above, for the final part of the study, a cohort of rats underwent transcardial perfusion with saline followed by glyoxal prior to immersion fixation.

*Retinal wholemount study and cryosection study*: For eyes to be used as retinal wholemounts or cryosections, the following fixatives were tested: (A) freshly prepared 4% PFA in phosphate buffer, pH 7.4. PFA eyes for the wholemount study were fixed for 1 h, and PFA eyes for the cryosection study were fixed for 2 h. (B) 3% glyoxal containing 0.8% acetic acid and 20% ethanol, pH 5. Glyoxal was prepared fresh, as described by Richter et al.^11^. In brief, for 100 ml solution, 70.9 ml ddH_2_O, 19.7 ml absolute ethanol, 7.8 ml glyoxal (40% stock solution from Sigma, #128465), and 0.75 ml glacial acetic acid were mixed. The solution was then brought to pH 5.0 using NaOH. Glyoxal fixation results in soft, fragile tissue. Following some preliminary testing, all glyoxal eyes for the wholemount study were fixed overnight (approximately 18 h). Glyoxal eyes for the cryosection study were fixed for either 2 h or overnight. Subsequently, a variation of glyoxal fixative (glyoxal-v), comprising 9% glyoxal/8% acetic acid, pH 4.0, as described by Konno et al.^13^, was tested in wholemounts and cryosections. Glyoxal-v eyes for both the wholemount and cryosection studies were fixed either for 2 h or overnight.

*Paraffin study*: For eyes to be embedded in paraffin, the following fixatives were tested: (A) 10% NBF; (B) Davidson’s solution, comprising 2 parts formaldehyde (37%), 3 parts 100% ethanol, 1 part glacial acetic acid and 3 parts water; (C) 3% glyoxal containing 0.8% acetic acid and 20% ethanol, pH 5.0. All eyes for the paraffin study were fixed overnight. Subsequently, the variation of glyoxal fixative (glyoxal-v) was tested. Glyoxal-v eyes for the paraffin study were fixed either for 2 h or overnight.

*Study design*: For the *wholemount study*, a total of 58 eyes were used. Initially, 2 h fixation with glyoxal (2 h; *n* = 4) was tested. Subsequently, a detailed comparison was made between PFA (*n* = 18 eyes) and overnight glyoxal fixation (*n* = 18 eyes). Next, variations of glyoxal, namely glyoxal-v (2 h; *n* = 9) and glyoxal-v (24 h; *n* = 9) were tested. For the *cryosection study*, a total of 12 eyes were used, which comprised 4 groups each of *n* = 3. The fixative groups were as follows: PFA, glyoxal (2 h), glyoxal (overnight), glyoxal-v (2 h). For the *paraffin study*, a total of 15 eyes were used, which comprised 5 groups (fixation regimes) each of *n* = 3. The fixative groups were as follows: NBF, Davidson’s, glyoxal, glyoxal-v (overnight), glyoxal-v (2 h). For the *LPS study*, a total of 9 eyes were used, which comprised 3 groups, each of *n* = 3. The fixative groups were as follows: NBF, Davidson’s, glyoxal. For the *laser injury study*, a total of 9 eyes were used, which comprised 3 groups, each of *n* = 3. The fixative groups were as follows: NBF, Davidson’s, glyoxal. For the *immersion versus perfusion study*, a total of 18 eyes were used as follows: paraffin experiment (*n* = 3 immersion; *n* = 3 perfusion); cryosection experiment (*n* = 3 immersion; *n* = 3 perfusion; wholemount experiment (*n* = 4 immersion; *n* = 4 perfusion).

## Tissue processing and histology

*Wholemount study*: Following fixation, all eyes were transferred to PBS, dissected into posterior eye cups, and the retinas removed and prepared as flattened wholemounts.

*Cryosection study*: Following fixation, all eyes were dissected into posterior eye cups, immersed in 15% sucrose in PBS for 2 h, and subsequently in 30% sucrose in PBS at 4 °C overnight. The next day, eye cups were embedded in optimal cutting temperature (OCT) compound (Sakura Finetek USA) and 6 μm transverse sections were cut using a cryostat (Leica CM3050 S). Sections were captured on Flex slides and stored at −20 °C until used.

*Paraffin study*: Following fixation, all eyes were transferred to 70% ethanol until processing. Whole eyes were hand-processed according to the following schedule: 90% ethanol for 1 h, 3 × 100% ethanol for 1 h, 2 x xylene for 1 h, 50% xylene/50% wax (Surgipath Paraplast, Leica) for 1 h at 62 °C, 2 x wax for 1 h at 62 °C, embed. Globes were embedded sagitally and 4 μm sections were cut using a rotary microtome. Sections were captured on Flex slides (Agilent, #K8020), blotted and incubated at 37 °C overnight before storage at room temperature in the dark. Tissue sections were stained for haematoxylin and eosin (H&E) using a standard methodology.

## Immunohistochemistry

*Paraffin study*: Colourimetric immunohistochemistry was performed essentially as previously described^5,15^. In brief, tissue sections were deparaffinised, rinsed in 100% ethanol and treated with 0.5% H_2_O_2_ in absolute methanol to block endogenous peroxidase activity before being taken to PBS. For localisation of certain antigens (see Table 2), antigen retrieval was not required. For the majority of antigens, heat-induced antigen retrieval (HIAR) was achieved by microwaving the sections in 10 mM citrate buffer, pH 6.0 for 10 min at 95–100 °C. For comparative purposes, some antibodies underwent HIAR using 100 mM Tris-HCl buffer, pH 9.0 (See Supplementary Table 4). For localisation of several antigens (see Table 2), sections received an additional enzyme digestion for 10 min with proteinase K (Merck-Millipore; 20 µg/ml at room temperature). Following antigen retrieval, tissue sections were rinsed in PBS, blocked in PBS containing 3% normal horse serum, then incubated overnight at room temperature in primary antibody (containing 3% normal horse serum; see Supplementary Table 1), followed by consecutive incubations with biotinylated secondary antibody (1:250; Vector) and streptavidin-peroxidase conjugate (1:1000; Pierce). Colour development was achieved using 3,3’-diaminobenzidine or Vector VIP substrate kit (Vector). Sections were counterstained with haematoxylin, dehydrated, cleared in xylene and mounted in DPX. Images of labeled sections were captured using a standard light microscope (BX51; Olympus, Mount Waverly, Victoria, Australia) with an attached vibration-free camera.

**Table 1:** Summary of compatibility of antibodies with PFA- and glyoxal-fixed, retinal cryosections.

Target	-------- Labelling intensity --------		Target	-------- Labelling intensity --------	
	PFA	glyoxal		PFA	glyoxal
CD11b	++/+++	++	GFAP	+++	+++
cone arrestin	+++	++	glutamine synthetase	+++	+++
FGF-2	++	-	isolectin-B4	+++	+++
glutaminase	+++	-	laminin	+++	+++
Hsp27	+++	+	M/L-opsin	++/+++	++/+++
Iba (WAKO)	++/+++	+	np-NFH	+++	+++
Iba1 (Novus)	++/+++	+/++	occludin-1	+++	+++
MCT1	+++	++/+++	PKC	+++	+++
nestin	+++	++	RBPMS	+++	+++
NFL	+++	++	rhodopsin	+++	+++
parvalbumin	+++	+	RPE65	+++	+++
peanut agglutinin	+++	++	syntaxin-1	+++	+++
β_3_-tubulin	+++	++	vimentin	+++	+++
aquaporin-4	+++	+++	ZO-1	+++	+++
calretinin	+++	+++	calbindin	++	+++
CD31	++	++	claudin-5	++	+++
CKMT1 A	+++	+++	Hsp70	++	+++

Note: PFA and glyoxal eyes were fixed for 2 h. Grading scheme: - = minimal specific labelling; + = weak specific labelling; ++ = modest specific labelling; +++ = intense specific labelling.

*Wholemount study*: Retinal wholemounts were incubated in 1% PBS-T for 1 h at room temperature, followed by 1% PBS-T containing 3% normal horse serum (NHS-T) for 1 h at room temperature to block non-specific binding. Retinas were then incubated for 3 days at 4 °C in primary antibody combinations. Subsequently, wholemounts were washed for 3 × 30 min in 1% PBS-T and incubated overnight at 4 °C with a combination of AlexaFluor-488 and − 594 conjugated secondary antibodies (1:750; Invitrogen). On the final day, wholemounts were washed for 3 × 30 min in PBS and mounted onto glass slides and coverslipped.

*Cryosection study*: Tissue sections were thawed at room temperature for 20 min, rinsed in PBS, incubated in 1% PBS-T (PBS containing 1% Triton X-100) for 20 min, rinsed in PBS, blocked in PBS containing 3% normal horse serum, then incubated overnight at room temperature in primary antibody (containing 3% normal horse serum; see Supplementary Table 1), followed by consecutive incubations with biotinylated secondary antibody (1:250; Vector) and streptavidin-conjugated AlexaFluor 594 (1:1000; Invitrogen). Sections were then counterstained with DAPI and mounted using anti-fade mounting medium. Images of labeled sections were acquired using a Zeiss Axio Scan.Z1 Digital Slide Scanner (Carl Zeiss).

Specificity of immunohistochemical labelling was judged by the morphology and distribution of the labelled cells, by the absence of signal when the primary antibody was replaced by isotype/serum controls, and by comparison with the expected staining pattern based on our own, and other, previously published results.

For each part of the study, and for each antibody tested, a semi-quantitative grading scheme was used to evaluate the immunostaining: - = minimal or lack of labelling; + = weak labelling; ++ = modest signal-to-background labelling; +++ = high signal-to-background labelling. For retinal wholemounts, a semi-quantitative grading scheme was used to evaluate the effect of each fixative regime on the structure of the dissected tissue: - = minimal damage to wholemount; + = occasional or minor tears/holes; ++ = multiple tears/holes; +++ = fragmented/badly torn retina. All analyses were conducted in a blinded fashion. For the cryosection study and paraffin study, all samples per group were analysed for each antibody. For antibodies where consistent, satisfactory labelling was obtained within the group, the antibody labelling was not repeated. For antibodies where suboptimal labelling was obtained, the experiment was repeated with new tissue sections to verify the finding.

## Results

The present study included three investigations: retinal wholemounts, retinal cryosections and paraffin-embedded eyes, each of which play an important role in neuropathological research. Throughout this study, we used the glyoxal formula of Richter et al.^11^. Some antibodies yielded sub-optimal immunohistochemical signals with glyoxal fixation; thus, we additionally tested a variation of glyoxal fixative, as described by Konno et al.^13^ (see Materials and Methods).

### Retinal wholemounts

#### Technical considerations

**Fig. 1:**
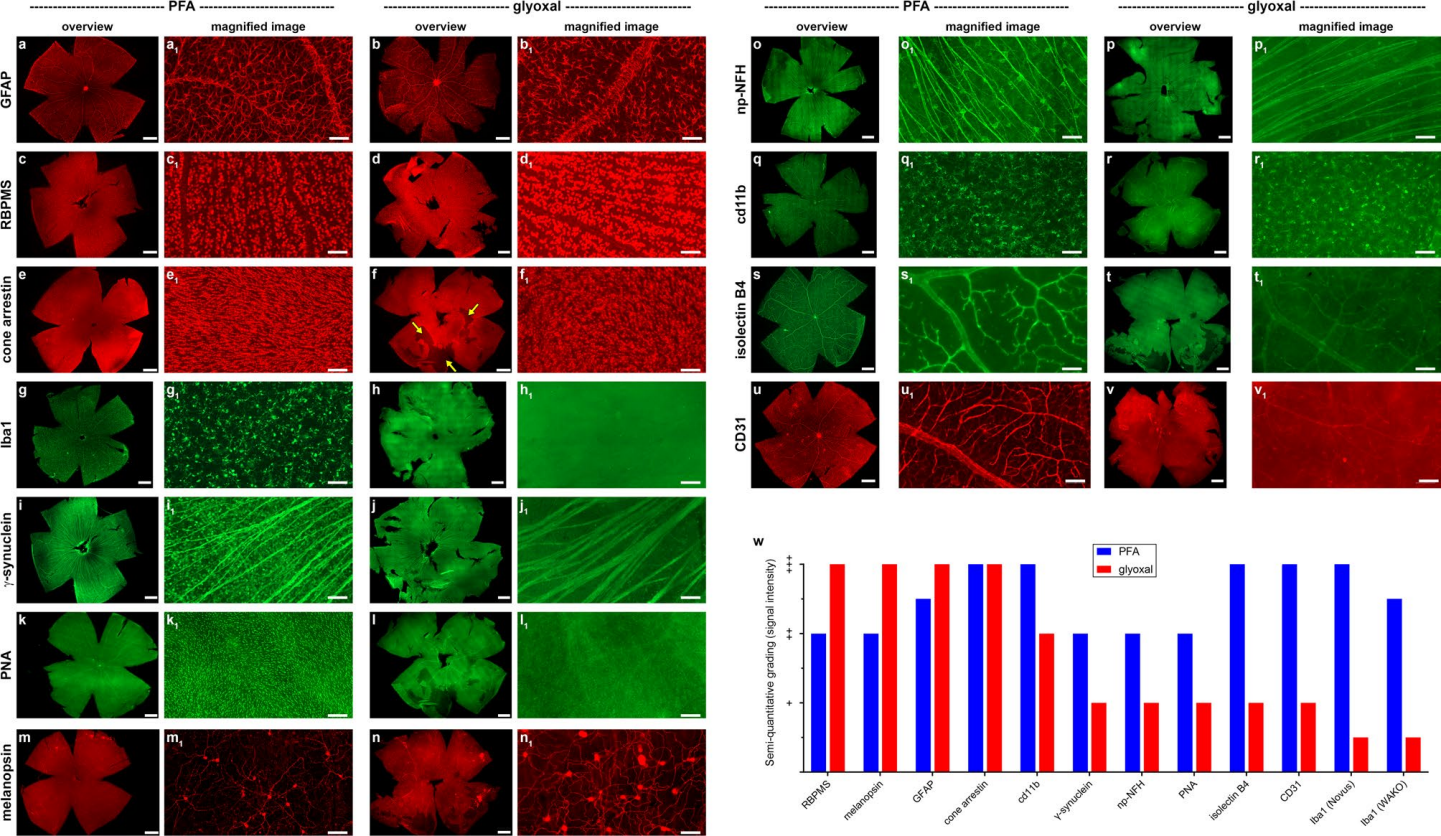
Representative images of neuronal and non-neuronal markers in PFA- and glyoxal-fixed wholemounts of retina, as delineated by fluorescent immunohistochemistry. (a, a_1_, b, b_1_) Astrocytes labelled by GFAP. (c, c_1_, d, d_1_) RGCs labelled by RBPMS. Immunoreactivity is more intense with glyoxal fixation. (e, e_1_, f, f_1_) Cone photoreceptor segments labelled by cone arrestin. Glyoxal fixation gently adheres the retina to the RPE and can result in patches of photoreceptor segments being lost during the wholemount dissection (arrows). (g, g_1_, h, h_1_) Microglia labelled by iba1. Iba1 is essentially non-reactive with glyoxal fixation. (i, i_1_, j, j_1_) RGC somas and their axons labelled by χ-synuclein. Signal-to-background labelling is weaker with glyoxal fixation. (k, k_1_, l, l_1_) Cone photoreceptor segments labelled by peanut agglutinin (PNA). PNA is only weakly reactive with glyoxal fixation. (m, m_1_, n, n_1_) Neurons labelled by melanopsin. Immunoreactivity is somewhat more intense with glyoxal fixation. (o, o_1_, p, p_1_) RGCs and their axons labelled by SMI-32 (np-NFH). Immunoreactivity is more intense with PFA fixation. (q, q_1_, r, r_1_) Microglia labelled by CD11b. Iba1 is more intense with PFA fixation. (s, s_1_, t, t_1_) Blood vessels labelled by isolectin B4. Signal-to-background labelling is substantially weaker with glyoxal fixation. (u, u_1_, v, v_1_) Blood vessels labelled by CD31. Signal-to-background labelling is substantially weaker with glyoxal fixation. (w) Immunohistochemical grading scheme: - = minimal specific labelling; + = weak specific labelling; ++ = modest specific labelling; +++ = intense specific labelling.

Immunolabelling of retinal wholemounts is a vital methodology for evaluating neurodegeneration. We compared immunoreactivities for some of the more commonly used markers in retinal research in glyoxal-fixed eyes versus eyes fixed for 1 h in 4% PFA, a standard fixation regime for retinal wholemounts. Immunolabelling of wholemounts is more demanding than immunolabelling of thin tissue sections. Various strategies have been employed to increase antibody penetration and signal intensity in wholemounts, for example, the use of long incubation times, inclusion of Triton-X100 in antibody solutions, and the addition of a freeze-thaw step. For comparative purposes, we utilised the same protocol for all antibodies and fixatives, which included 1% Triton-X100 in all incubation steps and a 3-day duration with primary antibody. Before considering immunoreactivities, however, there are two important technical considerations. PFA-fixed retinas were sufficiently robust that careful dissection from the eye cup produced intact, undamaged wholemounts (Supplementary Fig. 1a, f). Glyoxal-fixed retinas, in contrast, were soft and fragile. Fixation for short durations of 1–2 h presented an insurmountable challenge in terms of dissecting an undamaged retinal wholemount (Supplementary Fig. 1b, f). Even fixation overnight presented a genuine challenge, such that the majority of glyoxal-fixed wholemounts featured some artefactual tears and holes (Supplementary Fig. 1c, f; Fig. 1). A second technical consideration relates to separating the retina from the underlying RPE. This is straightforward in PFA-fixed eyes. In glyoxal-fixed eyes, however, the ethanol component of the fixative causes photoreceptor segments to adhere to the RPE^16^. While the retina can be separated in an ostensibly satisfactory manner, subsequent immunolabelling of the wholemount for markers of photoreceptor outer segments typically reveals small patches of retina that are denuded of segments (see Fig. 1).

#### Neuronal, glial and vascular markers

GFAP primarily demarcates astrocytes in the healthy retina. Both PFA and glyoxal produced excellent GFAP signal intensity in wholemounts (Fig. 1a, b, w), with the latter fixative resulting in slightly higher signal intensity. RBPMS labels the entire population of RGCs. PFA fixation elicited good signal intensity, while glyoxal fixation produced excellent signal intensity in wholemounts (Fig. 1c, d, w). Cone arrestin labels cone photoreceptor somata, axons and segments, although only the segments are visible in wholemounts from healthy retinas. Both PFA and glyoxal produced excellent cone arrestin signal intensity in wholemounts (Fig. 1e, f, w). In the healthy retina, Iba1 labels the entire microglial population. PFA fixation elicited excellent signal intensity, but Iba1 is essentially non-reactive with glyoxal fixation (Fig. 1g, h, w). This result was verified using two different Iba antibodies. χ-Synuclein labels RGC somas and their axons in the nerve fiber layer. PFA fixation elicited good χ-synuclein signal intensity, but labelling was weak and unsatisfactory with glyoxal fixation, especially of somas (Fig. 1i, j, w). Peanut agglutinin (PNA) is one of the most widely used markers of cone photoreceptor outer segments. As with χ-synuclein, good intensity labelling was evident after PFA fixation, but signal-to-noise was inadequate with glyoxal fixation (Fig. 1k, l, w). Melanopsin is a marker of intrinsically-photosensitive RGCs. PFA fixation elicited good melanopsin signal intensity in wholemounts, while glyoxal fixation produced excellent and improved signal intensity (Fig. 1m, n, w). SMI-32, a monoclonal antibody targeted against np-NFH, demarcates a subpopulation of RGCs. PFA fixation elicited satisfactory labelling of RGC somata and their axons in the nerve fiber layer. Glyoxal fixation, in contrast, elicited weak labelling of both somata and axons (Fig. 1o, p, w). Like Iba1, CD11b labels the entire microglial population in the retina. PFA fixation elicited good CD11b signal intensity with demarcation of fine processes, but the signal-to-background produced by glyoxal fixation was somewhat less satisfactory (Fig. 1q, r, w). Finally, we labelled for the vascular markers isolectin B4 and CD31, which produced excellent signals in PFA-fixed tissue, but a weak, inadequate signal in glyoxal-fixed eyes (Fig. 1s-w).

Overall, the results from the panel of markers tested on wholemounts revealed that no fixative was uniformly superior. Whilst three antibodies labelled better in glyoxal-fixed tissue, namely GFAP, RBPMS and melanopsin, more antibodies produced signal intensities that were not fit for purpose. In contrast, PFA produced good to excellent labelling for all eleven antibodies evaluated (Fig. 1w).

Testing of the glyoxal variation (glyoxal-v), which contains a 3-fold higher concentration of glyoxal and 10-fold higher concentration of acetic acid, did not provide better outcomes, irrespective of whether eyes were fixed for 2 h or overnight. Although these retinas, particularly those fixed overnight, were physically more robust than after conventional glyoxal fixation (Supplementary Fig. 1 d-f), they were still more fragile than after PFA fixation, and, importantly, signal intensities after glyoxal-v fixation were inferior for all antibodies tested as compared to glyoxal (Supplementary Table 2).

## Cryosections

Cryosections are routinely used in retinal research for reasons that relate primarily to preservation of antigenicity, practicality and cost. In a side-by-side comparison, we tested a panel of neuronal and non-neuronal antibodies in cryosections prepared from eyes fixed for 2 h in glyoxal versus eyes fixed for 2 h in 4% PFA, the conventional protocol used in retinal research. An incubation step with Triton X-100 was included for all antibodies, since it has been shown to be required for glyoxal-based immunohistochemistry using brain cryosections.

### Neuronal markers

**Fig. 2:**
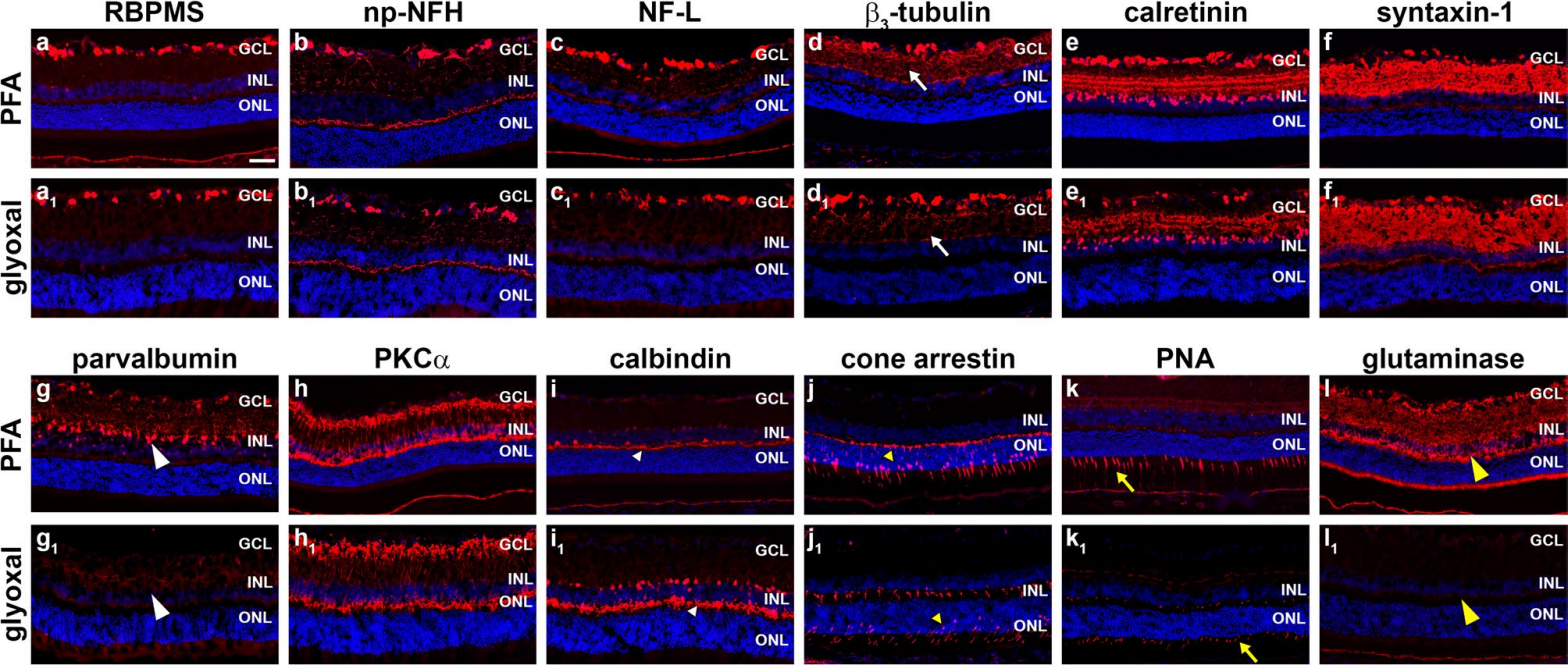
Representative images of neuronal markers in PFA- and glyoxal-fixed cryosections of retina, as delineated by fluorescent immunohistochemistry. All eyes were fixed for 2 h. (a, a_1_) RGCs labelled by RBPMS. (b, b_1_) RGC axon bundles and dendrites labelled by np-NFH. (c, c_1_) RGC axon bundles and dendrites labelled by NF-L. Immunoreactivity is slightly weaker with glyoxal fixation (white arrows). (d, d_1_) RGC somata, dendrites and axon bundles labelled by β_3_-tubulin. Immunoreactivity is somewhat weaker with glyoxal fixation (white arrows). (e, e_1_) Amacrine cells and RGCs labelled by calretinin with 3 layers of terminals visible. (f, f_1_) Amacrine cells and their processes labelled by syntaxin-1. (g, g_1_) Amacrine cells and their processes labelled by parvalbumin. Immunoreactivity is markedly weaker with glyoxal fixation (large white arrowheads). (h, h_1_) Rod bipolar cells and their processes terminating in the inner and outer plexiform layers labelled by PKCα. (i, i_1_) Horizontal cells and their processes labelled by calbindin. Immunoreactivity is more intense with glyoxal fixation (small white arrowheads). (j, j_1_) Cone photoreceptors labelled by cone arrestin. Immunoreactivity is slightly weaker with glyoxal fixation (small yellow arrowheads). (k, k_1_) Cone photoreceptor segments labelled by peanut agglutinin (PNA). Immunoreactivity is weaker with glyoxal fixation (yellow arrows). (l, l_1_) Expression of the neuronal marker glutaminase. Immunoreactivity is almost undetectable with glyoxal fixation (large yellow arrowheads). Scale bar: 50 μm. GCL, ganglion cell layer; INL, inner nuclear layer; ONL, outer nuclear layer.

Of the RGC markers employed, RBPMS and np-NFH labelled with broadly similar intensities in PFA- and glyoxal-fixed sections (Fig. 2a, b; Table 1), while NFL and β_3_-tubulin were somewhat stronger in PFA-fixed tissue than glyoxal-fixed tissue (Fig. 2c d; Table 1). Immunoreactivities for the amacrine cell markers calretinin (Fig. 2e; Table 1) and syntaxin-1 (Fig. 2f; Table 1) were each of high intensity for both fixatives, however, parvalbumin was substantially weaker with glyoxal fixation (Fig. 2g; Table 1). Immunoreactivities for the rod bipolar marker PKCα (Fig. 2h; Table 1), the rod photoreceptor marker rhodopsin (Supplementary Fig. 2a; Table 1), and the cone photoreceptor marker M/L-opsin (Supplementary Fig. 2b; Table 1) were each of high intensity irrespective of the fixative used. Glyoxal fixation resulted in stronger labelling for the horizontal cell marker calbindin compared to PFA fixation (Fig. 2i; Table 1). Conversely, glyoxal fixation elicited weaker labelling than PFA for the cone photoreceptor markers cone arrestin and PNA (Fig. 2j, k; Table 1). Labelling for the neuron-specific marker glutaminase was strong in PFA-fixed cryosections, but the antibody was effectively non-reactive after glyoxal fixation (Fig. 2l; Table 1). Finally, immunoreactivity for mitochondrial creatine kinase, which localizes to neuronal mitochondria, was strong with both fixatives (Supplementary Fig. 2c; Table 1).

**Table 2:** Summary of compatibility of antibodies with formalin-, Davidson’s- and glyoxal-fixed, paraffin-embedded retinas.

Target	------------ Labelling intensity ------------			Target	------------ Labelling intensity ------------		
	NBF	Davidson’s	glyoxal		NBF	Davidson’s	glyoxal
Brn3a	+++	++	++	np-NFH	+++	+++	+++
cone arrestin	+++	++/+++	++/+++	PKC	+++	+++	+++
glutaminase	+++	-/+	-/+	RBPMS	+++	+++	+++
NFL	+++	+++	++	synaptophysin	+++	+++	+++
parvalbumin	++/+++	+++	+	syntaxin-1	+++	+++	+++
PNA	+++	+++	+/++	vGLUT	+++	+++	+++
rhodopsin	+++	++/+++	++/+++	χ-synuclein	++	+++	+++
β_3_-tubulin	+++	+++	+	calbindin	++	++	+++
MAP2	+++	+++	+++^1^	calretinin	++	+++	+++^1^
melanopsin	+++	+++	+++				
iba1 (^a^ or ^b^)	+++	+	-/+	glutamine synthetase	+++	+++	+++
aquaporin-4	+++	+++	+++	S100	+++	+++	+++
GFAP	+++	+++	+++	vimentin	+++	+++	+++
claudin-5	+++^2^	+++	++^2^	laminin	+++^2^	+++^2^	+++^2^
CD31	++/+++	+++	++/+++	RPE65	+++	+++	+++
collagen VI	+++^2^	+++^2^	+++^2^	ZO-1	++^2^	++^2^	++^2^
isolectin-B4	+++	+++	+++				
cyclin D1	++	+	+	CD3	+++^2^	+++^2^	+++^3^
Hsp27	+++	++	+	CNTF	+++	+++	+++
Hsp70	+++	+	+	ED1	+++	++	+++
nestin	+++	++	++	IL-1β	+++^1^	+++	+++^1^
PCNA	+++	+++	-	MPX	+++	+++	+++^1^
PDH	++	++/+++	+	GLUT1	+++	+++	+++
CKMT1 A	+++	+++	+++	MCT1	+++	+++	+++
COX4	+++	+++	+++	PKM2	++	+++	++

Note: NBF (neutral buffered formalin), Davidson’s and glyoxal eyes were fixed overnight. ^a^Novus antibody; ^b^WAKO antibody^1^;No antigen retrieval required^2^;Combination of heat-induced and proteolytic-induced antigen retrieval required^3^;Proteolytic-induced antigen retrieval required. Grading scheme: - = minimal specific labelling; + = weak specific labelling; ++ = modest specific labelling; +++ = intense specific labelling.

### Non-neuronal markers

**Fig. 3:**
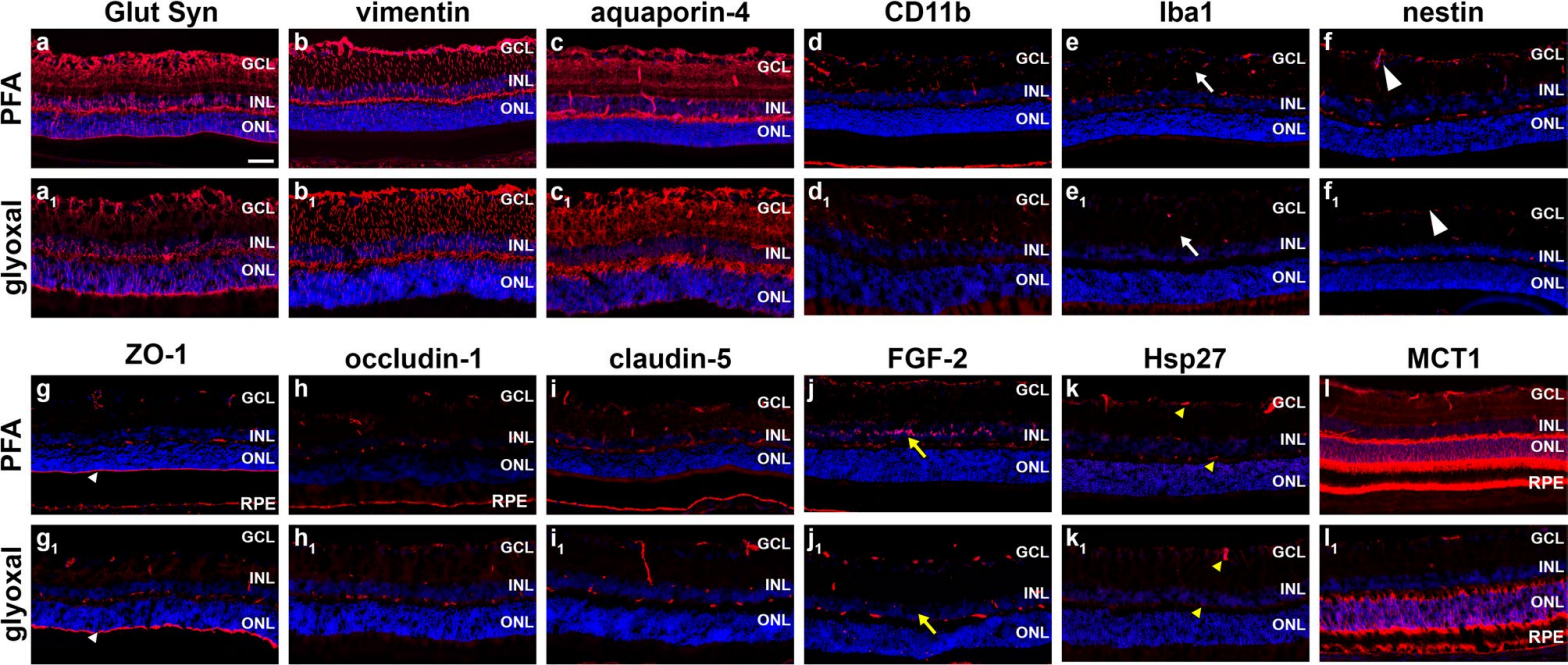
Representative images of non-neuronal markers in PFA- and glyoxal-fixed cryosections of retina, as delineated by fluorescent immunohistochemistry. All eyes were fixed for 2 h. (a, a_1_) Macroglia (astrocytes and Müller cells) labelled by glutamine synthetase (Glut Syn). (b, b_1_) Astrocytes and Müller cell processes labelled by vimentin. (c, c_1_) Macroglia and blood vessels labelled by aquaporin-4. (d, d_1_) Microglia labelled by iba1. Many microglia are undetectable with glyoxal fixation (white arrows). (e, e_1_) Microglia labelled by CD11b. Immunoreactivity is slightly weaker with glyoxal fixation. (f, f_1_) Blood vessels and Müller cell endfeet labelled by nestin. Immunoreactivity is weaker with glyoxal fixation (large white arrowheads). (g, g_1_) Tight junctions forming the blood-retinal barrier (blood vessels and RPE) plus the outer limiting membrane (small white arrowheads) labelled by ZO-1. (h, h_1_) Tight junctions forming the blood-retinal barrier (blood vessels and RPE) labelled by occludin-1. (i, i_1_) Tight junctions forming the blood-retinal barrier (blood vessels and RPE) labelled by claudin-5. Immunoreactivity is stronger with glyoxal fixation (j, j_1_) Müller cell somas labelled by FGF-2. No specific labelling is detectable with glyoxal fixation (yellow arrows). Non-specific labelling of blood vessels is evident. (k, k_1_) Blood vessels and some astrocytes (yellow arrowheads) labelled by the inducible heat shock protein Hsp27. Immunoreactivity is markedly weaker with glyoxal fixation. (l, l_1_) Blood vessels, photoreceptors and the RPE labelled by the monocarboxylate transporter MCT1. Immunoreactivity is somewhat weaker with glyoxal fixation (white arrows). Scale bar: 50 μm. GCL, ganglion cell layer; INL, inner nuclear layer; ONL, outer nuclear layer; RPE, retinal pigment epithelium.

Antibodies directed against GFAP, vimentin (Fig. 3a) and glutamine synthetase (Fig. 3b), all of which label retinal macroglia, yielded similar high intensity immunoreactivities in PFA- and glyoxal-fixed tissues (Table 1). Immunoreactivity for aquaporin-4, which localises to retinal astrocytes, Müller cells and blood vessels, was also strong with both fixatives (Fig. 3c; Table 1). For identification of microglia, PFA produced slightly stronger immunolabelling with CD11b (Fig. 3d; Table 1), while Iba1 immunolabelling was unsatisfactory after glyoxal fixation, irrespective of whichever Iba primary antibody was employed (Fig. 3e; Table 1). In healthy retinas, nestin labels blood vessels and Müller cell endfeet. Nestin immunolabelling was satisfactory after PFA but not glyoxal fixation (Fig. 3f; Table 1). Six markers of the blood-retinal barrier, namely ZO-1, occludin-1, claudin-5, RPE65, laminin and isolectin-B4, all displayed good signal intensities with both fixatives (Fig. 3g-i; Supplementary Fig. 2 d; Table 1). CD31 produced adequate signal-to-background with both fixatives (Supplementary Fig. 2e; Table 1). The stress proteins FGF-2 (Fig. 3j; Table 1) and Hsp27 (Fig. 3k; Table 1) produced adequate signals in PFA-fixed retinas, but they elicited negligible or weak immunolabelling with glyoxal fixation. In contrast, Hsp70 elicited stronger labelling after glyoxal fixation (Supplementary Fig. 2f; Table 1). Furthermore, labelling for the monocarboxylate transporter MCT1 was also weaker with glyoxal fixation relative to PFA (Fig. 3l; Table 1).

### Testing of methodological alterations

The results from the panel of antibodies tested revealed that PFA fixation was generally superior to glyoxal for immunolabelling of cryosections (Table 1). To ascertain whether the immunolabelling protocol adopted for testing glyoxal was sub-optimal, we tested two methodological modifications: firstly, we fixed glyoxal eyes overnight – the protocol used by Konno et al.^13^ – rather than for 2 h. Secondly, we tested the glyoxal variation (glyoxal-v) that contains higher concentrations of glyoxal and acetic acid. Testing was generally focused on antibodies that did not provide high signal-to-background labelling with glyoxal fixation. In general, increasing the fixation time with glyoxal to overnight decreased antigenicity. Of the 15 antibodies tested, 8 displayed reduced immunoreactivity (Supplementary Fig. 3; Supplementary Table 3). Curiously, this was not the case for the microglial marker CD11b, which showed improved signal abundance compared to overnight fixation. A short duration of glyoxal fixation also failed to improve signal intensity for various other antibodies, including glutaminase, FGF-2, Hsp27 and parvalbumin. Given the overall dataset, it appears that PFA is more widely compatible than conventional glyoxal formula for immunolabelling of retinal cryosections.

Testing of the glyoxal variation (glyoxal-v) yielded mixed results (Supplementary Fig. 3; Supplementary Table 3). The morphology of the cryosections was outstanding, and, certain antibodies, such as CD31 and Hsp70 produced high signal intensities that were superior to those of glyoxal itself; however, other antibodies produced immunoreactivities no better than eyes fixed for 2 h in glyoxal, and some antibodies produced inferior immunolabelling to glyoxal. Importantly, both microglial markers, Iba1 and CD11b, were incompatible with glyoxal-v. Overall, glyoxal-v did not satisfactorily address the weaknesses of the conventional glyoxal formulation and, based on the large panel of antibodies that we employed, both formulations are less compatible than PFA for immunolabelling of retinal cryosections.

## Paraffin-embedded eyes

Paraffin-embedded eyes are routinely used for histopathology. They are suited to long term storage, the tissue block can be re-sectioned on multiple occasions without resulting in artefactual tissue damage, they produce more robust tissue sections, thinner sections can be cut from paraffin- than from cryo-embedded tissue, and they typically provide superior tissue morphology to cryosections. For this phase of the study, colorimetric rather than fluorescent immunolabelling was used.

*Morphology*: The morphology of NBF-fixed globes was generally adequate, but there were obvious deficiencies relative to Davidson’s- or glyoxal-fixed eyes: firstly, there was always some degree of processing-induced retinal detachment; secondly, the nuclear and plexiform layers were less compact and often featured artefactual tears; thirdly, cellular detail was inferior (Supplementary Fig. 4a, b). Davidson’s-fixed retinas exhibited outstanding morphological integrity with no artefactual detachment (Supplementary Fig. 4c). Glyoxal-fixed globes displayed better morphological integrity than NBF eyes and only minor retinal detachment (Supplementary Fig. 4 d).

### Optimization of immunohistochemistry

Immunohistochemical labelling of NBF- and Davidson’s-fixed, paraffin-embedded ocular sections typically requires heat-induced antigen retrieval (HIAR)^5,15^. Of the 49 antibodies – representing markers of neurons, macroglia, microglia, the blood-retinal barrier, inflammation, stress, and metabolism – tested on paraffin-embedded tissue in the current study, HIAR (in combination with proteolytic antigen retrieval for a small number of antibodies) was needed to produce good signal intensity for almost all of them (Table 2). The optimal conditions for performing immunohistochemistry on glyoxal-fixed retinas are unknown, but, published studies state that glyoxal-fixed tissues do not routinely need antigen retrieval^9,13^. Initially, therefore, we performed a side-by-side comparison of glyoxal-fixed tissue sections that received no retrieval versus tissue sections that underwent the standard HIAR method (10 mM citrate buffer, pH 6). The data showed that immunolabelling intensity was higher after HIAR for the majority of antibodies tested (See Supplementary Fig. 5 for representative images). For some antibodies, signal intensity was only modestly enhanced by HIAR, such as the RGC markers χ-synuclein and RBPMS. For other antibodies, including the axonal cytoskeleton proteins β_3_-tubulin and NFL, signal intensity was negligible without HIAR. Of importance, background labelling was not appreciably higher after standard HIAR. Accordingly, unless unwarranted, HIAR was used for all side-by-side comparison of glyoxal-fixed eyes with NBF- and Davidson’s-fixed eyes.

### Neuronal markers

**Fig. 4:**
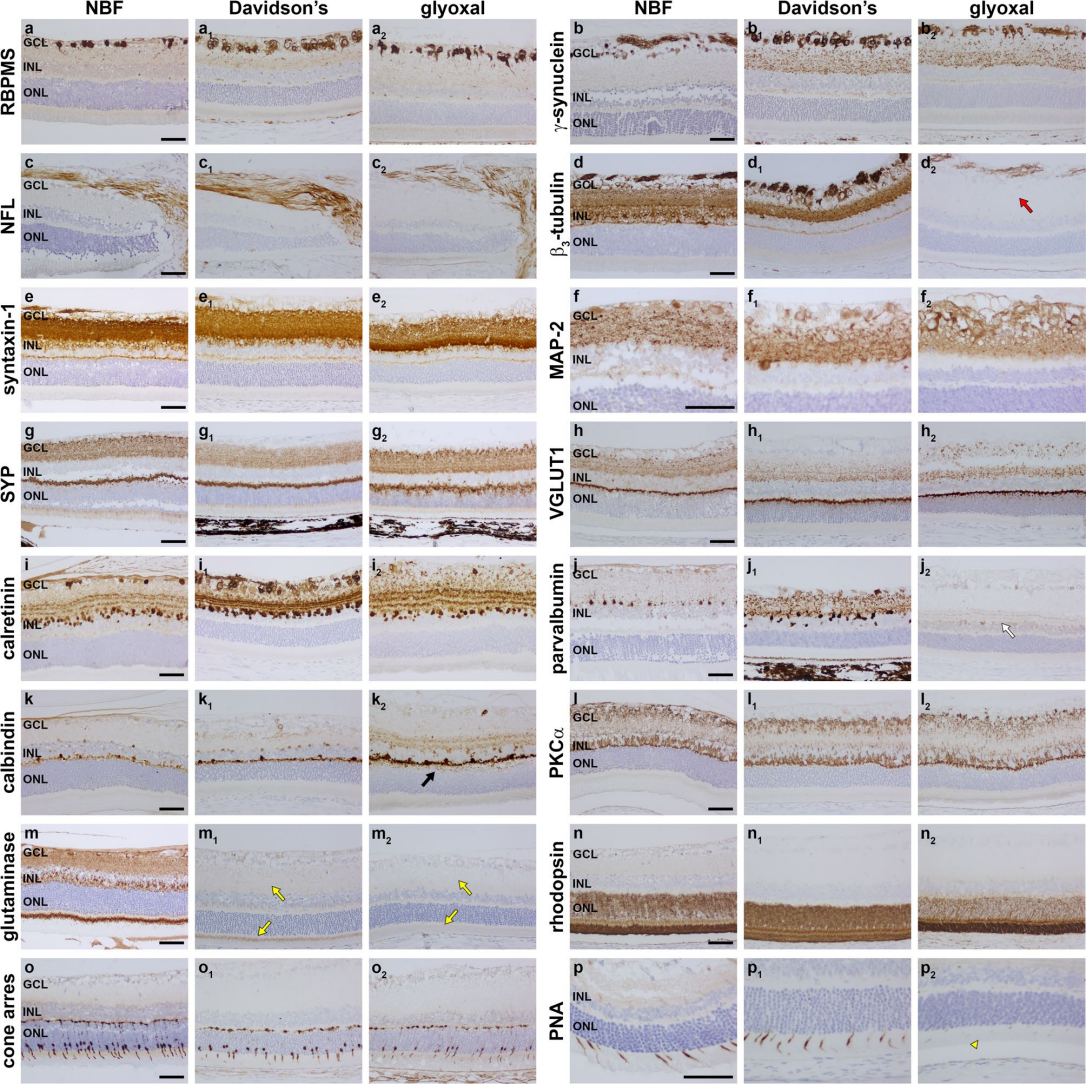
Representative images of neuronal markers in NBF-, Davidson’s- and glyoxal-fixed, paraffin-embedded sections of retina, as delineated by colorimetric immunohistochemistry. (a, a_1_, a_2_) RGCs labelled by RBPMS. (b, b_1_, b_2_) RGC somata, dendrites and axon bundles labelled by χ-synuclein. (c, c_1_, c_2_) RGC axons labelled by NFL. (d, d_1_, d_2_) RGC somata, dendrites and axon bundles labelled by β_3_-tubulin. Immunoreactivity is considerably weaker with glyoxal fixation (red arrow). (e, e_1_, e_2_) Amacrine cells and their processes labelled by syntaxin-1. (f, f_1_, f_2_) RGCs and their dendrites labelled by the microtubule-associated protein MAP-2. (g, g_1_, g_2_) Synaptic vesicles in the inner and outer plexiform layers labelled by synaptophysin. (h, h_1_, h_2_) Glutamatergic synapses in the inner and outer plexiform layers labelled by VGLUT1. (i, i_1_, i_2_) Amacrine cells and RGCs labelled by calretinin with 3 layers of terminals visible. (j, j_1_, j_2_) Amacrine cells and processes labelled by parvalbumin. Immunoreactivity is very weak with glyoxal fixation (white arrow). (k, k_1_, k_2_) Horizontal cells and their processes labelled by calbindin. Immunoreactivity is more intense with glyoxal fixation (black arrow). (l, l_1_, l_2_) Rod bipolar cells and their processes terminating in the inner and outer plexiform layers labelled by PKCα. (m, m_1_, m_2_) Expression of the neuronal marker glutaminase. Immunoreactivity is very weak with Davidson’s fixation and barely detectable with glyoxal fixation (yellow arrows). (n, n_1_, n_2_) Rod somata and segments labelled by rhodopsin. (o, o_1_, o_2_) Cone photoreceptor somata together with their axon terminals and segments labelled by cone arrestin. Immunoreactivity is slightly more intense with NBF (yellow arrowheads). (p, p_1_, p_2_) Cone photoreceptor segments labelled by peanut agglutinin (PNA). Immunoreactivity is markedly weaker with glyoxal fixation (yellow arrowhead). Scale bars: 60 μm. GCL, ganglion cell layer; INL, inner nuclear layer; ONL, outer nuclear layer.

We undertook a comprehensive evaluation of neuronal proteins in the retina, encompassing many markers used in studies investigating neuropathology and neurodegeneration in the Retina. Of the RGC markers employed, χ-synuclein labelling was optimal in Davidson’s and glyoxal-fixed sections but weaker after NBF fixation, RBPMS, melanopsin – which is restricted to intrinsically-photosensitive RGCs –, and np-NFH labelled with broadly similar intensities in PFA-, Davidson’s and glyoxal-fixed sections (Fig. 4a, b; Table 2), NFL and Brn3a immunoreactivities was somewhat weaker in glyoxal-fixed tissue (Fig. 4c; Table 2), while β_3_-tubulin immunoreactivity was considerably weaker with glyoxal fixation such that RGC processes were barely detectable (Fig. 4d Table 2). The amacrine cell marker syntaxin-1 produced strong labelling with all three fixatives (Fig. 4e; Table 2). Likewise, the dendritic protein MAP-2 and the synaptic vesicle-associated proteins synaptophysin and vGLUT1 were all highly compatible with each of the three fixatives (Fig. 4f-h; Table 2). The calcium binding proteins calretinin, parvalbumin and calbindin have distinct neuronal distributions in the rodent retina. Calretinin, which is expressed by subsets of amacrine cells and RGCs, was visualised equally well irrespective of the fixative used (Fig. 4i; Table 2). Parvalbumin displayed strongest immunolabelling after Davidson’s fixation, was adequate after NBF fixation, and barely detectable following glyoxal fixation (Fig. 4j; Table 2). In contrast, calbindin immunoreactivity, which is predominantly associated with horizontal cell somata and their dendrites, was satisfactory after NBF and Davidson’s fixation, but was more intense after glyoxal fixation (Fig. 4k; Table 2). The rod bipolar cell marker PKCα yielded a strong signal with all three fixatives (Fig. 4l; Table 2). Immunolabelling for the mitochondrial enzyme glutaminase, which is traditionally used to identify glutamate-releasing neurons, was intense after NBF fixation, but negligible after either Davidson’s or glyoxal fixation (Fig. 4m; Table 2). Rod and cone photoreceptors, identified by antibodies directed against rhodopsin and cone arrestin, respectively, were reliably immunolabelled whichever fixative was used, although highest signal intensity was obtained in combination with NBF (Fig. 4n, o; Table 2). As was the case in wholemounts and cryosections, PNA immunolabelling of cone photoreceptor outer segments was weak after glyoxal fixation (Fig. 4p; Table 2).

### Markers of glia and blood-retinal barrier

**Fig. 5:**
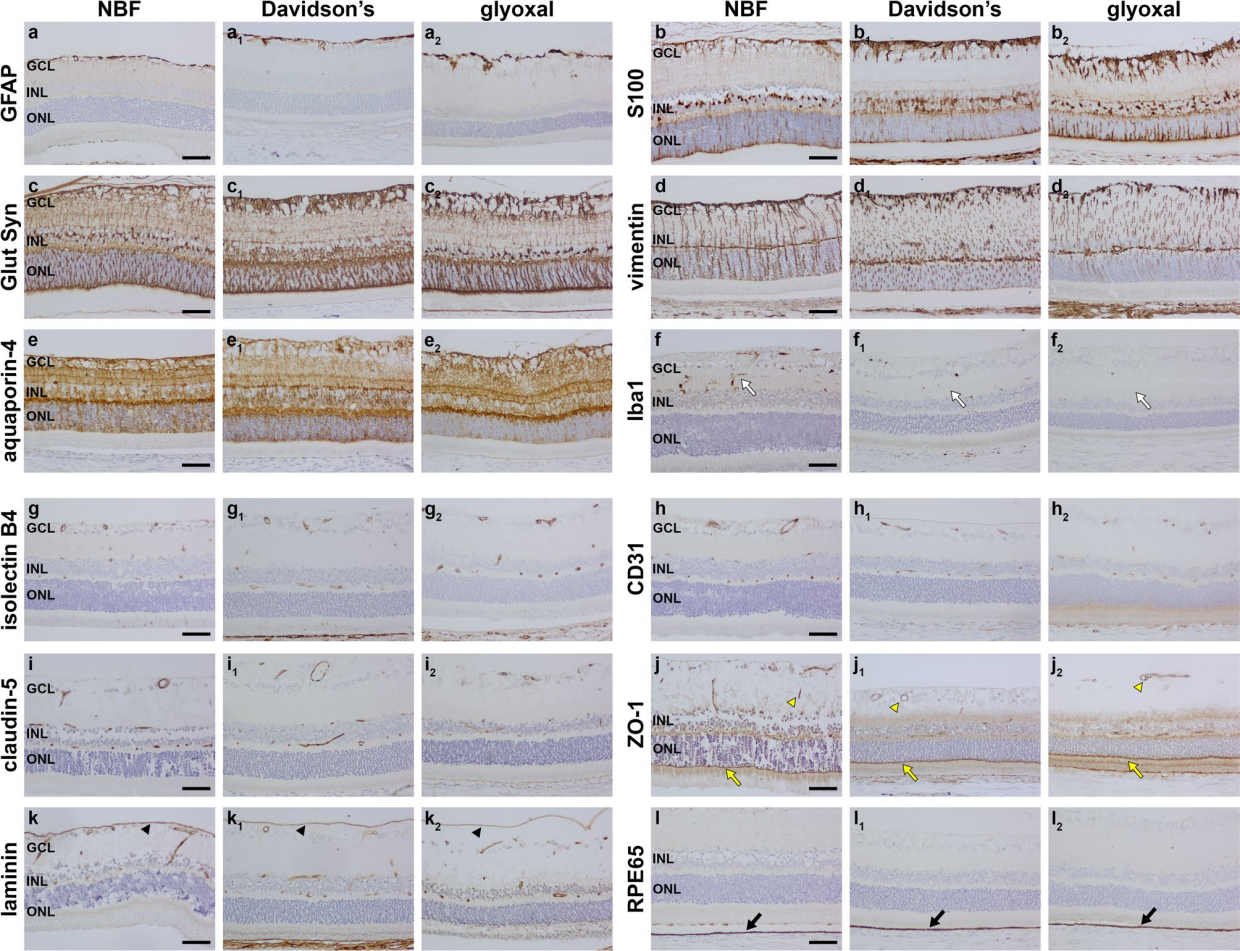
Representative images of glial markers and markers of the blood-retinal barrier in NBF-, Davidson’s- and glyoxal-fixed, paraffin-embedded sections of retina, as delineated by colorimetric immunohistochemistry. (a, a_1_, a_2_) Astrocytes labelled by GFAP. (b, b_1_, b_2_) Macroglia (astrocytes and Müller cells) labelled by S100. (c, c_1_, c_2_) Macroglia (astrocytes and Müller cells) labelled by glutamine synthetase (Glut Syn). (d, d_1_, d_2_) Astrocytes and Müller cell processes labelled by vimentin. (e, e_1_, e_2_) Macroglia and blood vessels labelled by aquaporin-4. (f, f_1_, f_2_) Microglia labelled by iba1. Compared with NBF eyes, microglia are weakly labelled in Davidson’s eyes and largely undetectable with glyoxal fixation (white arrows). (g, g_1_, g_2_) Blood vessels labelled by isolectin B4. (h, h_1_, h_2_) Blood vessels labelled by CD31. Macroglia (astrocytes and Müller cells) labelled by S100. (i, i_1_, i_2_) Tight junctions forming the blood-retinal barrier labelled by claudin-5. (j, j_1_, j_2_) Tight junctions forming the blood-retinal barrier (yellow arrowheads) plus the outer limiting membrane (yellow arrows) labelled by ZO-1. (k, k_1_, k_2_) The inner limiting membrane (black arrowheads) and blood vessels labelled by laminin. (l, l_1_, l_2_) The RPE labelled by RPE65 (black arrows). Scale bars: 60 μm. GCL, ganglion cell layer; INL, inner nuclear layer; ONL, outer nuclear layer.

Similar intensity and signal-to-background patterns of staining were observed in NBF- Davidson’s- and glyoxal-fixed tissues after incubation with antibodies to the macroglial markers GFAP, S100, glutamine synthetase and vimentin (Fig. 5a-d; Table 2). Aquaporin-4 immunolabelling of macroglia and blood vessels was also robust in tissues treated with each of the three fixatives (Fig. 5e; Table 2). In contrast, while Iba1 immunolabelling was abundant and well-defined in NFB-fixed retinas, labelling was weak in Davidson’s-fixed sections, and negligible in glyoxal-fixed tissue (Fig. 5f; Table 2). The lack of compatibility of glyoxal with Iba1 immunolabelling was consistent across both primary antibodies evaluated and closely matched data from wholemounts and cryosections.

Regarding the blood-retinal-barrier, the vascular marker isolectin B4 (Fig. 5g; Table 2) was visualised equally well whichever fixative was used, while antibodies to CD31 and claudin-5 were also reactive in tissue sections prepared from all three fixatives but displayed slightly lower signal-to-background labelling with glyoxal fixation (Fig. 5h, i; Table 2). The antibody targeted to ZO-1 was less suited to paraffin-embedded tissue than frozen tissue, but yielded specific labelling of tight junctions in NBF, Davidson’s and glyoxal-fixed eyes, albeit with concurrent background staining in each case (Fig. 5j; Table 2). Antibodies directed against the extracellular matrix proteins collagen VI (Supplementary Fig. 2 d; Table 2) and laminin (Fig. 5k; Table 2).

elicited satisfactory labelling with all three fixatives, as did the antibody targeted to RPE65, the well-established marker of retinal pigment epithelium (RPE) cells (Fig. 5l; Table 2).

### Markers of inflammation and stress

**Fig. 6:**
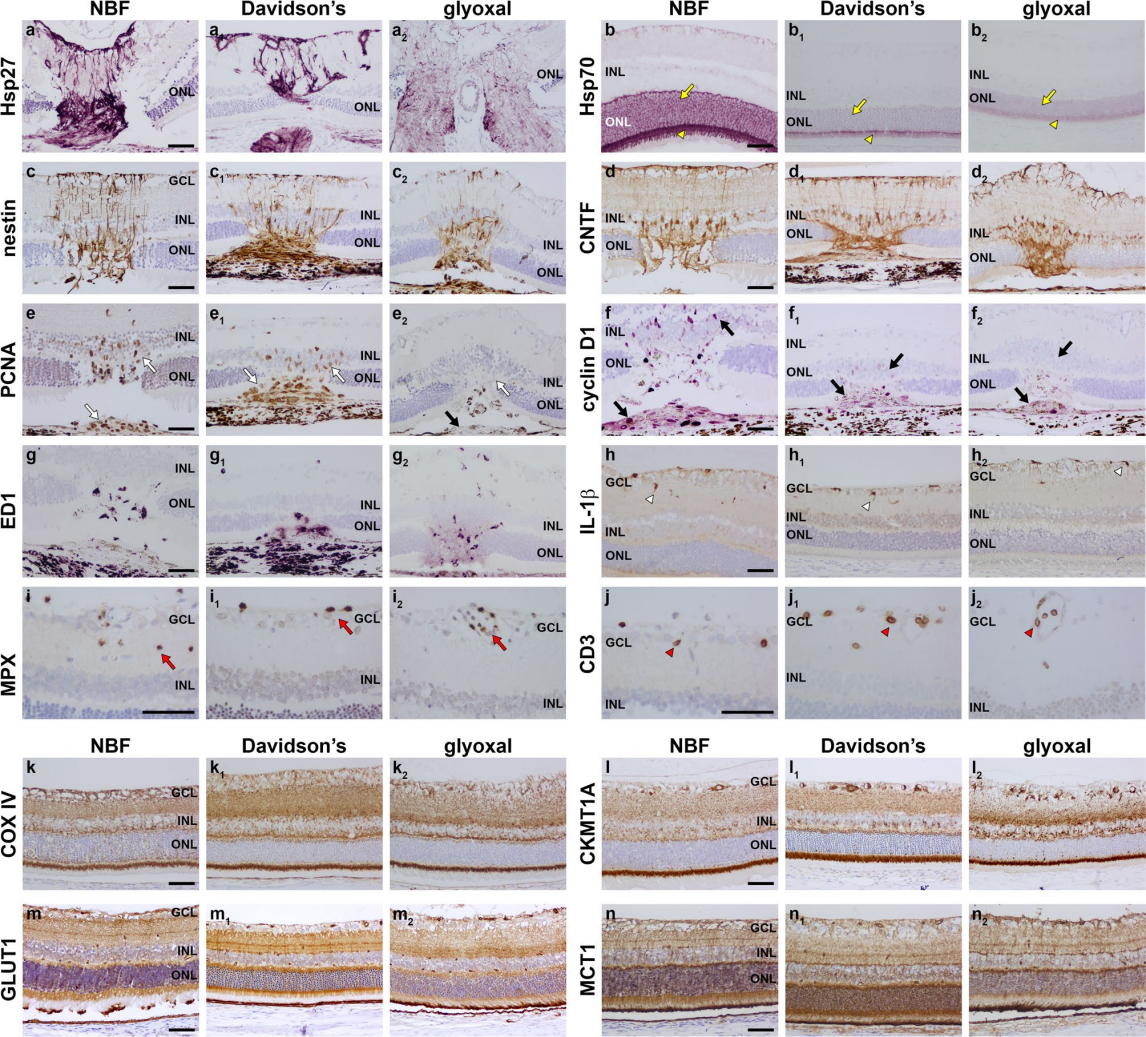
Representative images of markers of cellular stress, inflammation and bioenergetics in NBF-, Davidson’s- and glyoxal-fixed, paraffin-embedded sections of retina, as delineated by colorimetric immunohistochemistry. (a, a_1_, a_2_) Astrocytes at the optic nerve head labelled by the inducible heat shock protein Hsp27. Compared with NBF eyes, immunolabellling is slightly weak in Davidson’s-fixed eyes and substantially weaker in glyoxal-fixed eyes. (b, b_1_, b_2_) Photoreceptor somata (yellow arrows) and segments (yellow arrowheads) labelled by the inducible heat shock protein Hsp70. Compared with NBF eyes, immunolabellling is weak in Davidson’s- and glyoxal-fixed eyes. (c, c_1_, c_2_) Nestin expression at the site of laser lesion. Immunoreactivity is weaker with glyoxal fixation. (d, d_1_, d_2_) CNTF expression at the site of laser lesion. (e, e_1_, e_2_) PCNA-positive cells at the site of laser lesion (white arrows). PCNA is non-reactive with glyoxal fixation. (f, f_1_, f_2_) Cyclin D1-positive cells at the site of laser lesion (black arrows). Compared with NBF eyes, immunolabellling is weaker in Davidson’s- and glyoxal-fixed eyes. (g, g_1_, g_2_) ED1-positive cells at the site of laser lesion. (h, h_1_, h_2_) IL-1β-positive microglia in the nerve fibre and inner plexiform layers following LPS treatment (white arrowheads). (i, i_1_, i_2_) Neutrophils labelled by myeloperoxidase (MPX) following LPS treatment (red arrows). (j, j_1_, j_2_) T cells labelled by CD3 following LPS treatment (red arrowheads). (k, k_1_, k_2_) Mitochondria labelled by cytochrome C oxidase (COX IV). (l, l_1_, l_2_) Mitochondria labelled by ubiquitous mitochondrial creatine kinase subunit 1 A (CKMT1 A). (m, m_1_, m_2_) Expression of Glucose transport-1 (GLUT1). (n, n_1_, n_2_) Expression of the monocarboxylate transporter-1 (MCT1). Scale bars: 60 μm. GCL, ganglion cell layer; INL, inner nuclear layer; ONL, outer nuclear layer.

Heat shock proteins (Hsps) are a family of molecular chaperones and cellular stress response proteins. In normal retinas, astrocytes constitutively express Hsp27, while photoreceptors express Hsp70^17^. Our previous work has shown that antibodies to both Hsps are less reactive in Davidson’s-fixed retinas than those fixed in NBF^5^. The current results confirm this finding and reveal that glyoxal fixation is even less suited to visualization of Hsp27 and 70 in paraffin-embedded tissue (Fig. 6a, b; Table 2), data that correspond with results from the cryosection phase of this study.

To investigate localization of certain glial stress proteins and infiltrating leukocytes we evaluated retinas from eyes treated with either the bacterial toxin LPS, or with argon laser, which causes localized thermal injury to the photoreceptors. Following laser injury, the intermediate filament nestin and the neurotrophic factor ciliary neurotrophic factor (CNTF) are expressed in scar tissue at the site of lesion and in overlying Müller cell processes^18^. Compared with NBF-fixed eyes, nestin immunolabelling in Davidson’s- and glyoxal-fixed eyes was weaker (Fig. 6c; Table 2). Ciliary neurotrophic factor (CNTF), in contrast, displayed similar intensity labelling in retinas from all three fixatives (Fig. 6d; Table 2). To identify glial cell de-differentiation or proliferation, we tested antibodies against proliferating cell nuclear antigen (PCNA) and cyclin D1. In NBF-fixed tissue, PCNA- and cyclin D1-positive cells were readily identified overlying laser lesions (Fig. 6e, F; Table 2). In Davidson’s-fixed retinas, PCNA labelling was satisfactory, but cyclin D1 signal intensity was weak (Fig. 6e, f; Table 2). In glyoxal-fixed retinas, the PCNA antibody was essentially non-reactive and cyclin D1 signal intensity was weak (Fig. 6e, f; Table 2). ED1-positive microglia/macrophages were readily identifiable in tissue sections from all three fixatives (Fig. 6g; Table 2). The proinflammatory cytokine interleukin-1β (IL-1β) localizes to a subset of microglia in the inner retina after injection of LPS^5^. IL-1β immunolabelling was equally compatible with all three fixatives (Fig. 6h; Table 2). Lastly, we evaluated the presence of infiltrating neutrophils and T cells after LPS treatment using antibodies directed against myeloperoxidase and CD3, respectively. Satisfactory labelling was obtained for both markers with each of the three fixatives (Fig. 6i, j; Table 2).

### Markers of energy metabolism

Antibodies directed against cytochrome C oxidase (COX IV) and mitochondrial creatine kinase (CKMT1 A) elicited intense labelling with all three fixatives (Fig. 6k, l; Table 2). This was also the case for the glucose and monocarboxylate transporters GLUT1 and MCT1 (Fig. 6m, n; Table 2), although labelling for each of these proteins was more clearly delineated in retinas fixed in Davidson’s or glyoxal due to their better morphology. The mitochondrial enzyme pyruvate dehydrogenase (PDH) was visualised with high signal-to-background after Davidson’s fixation, but was less satisfactory after NBF and particularly glyoxal fixation (Supplementary Fig. 2e; Table 2). Finally, the glycolytic isoenzyme pyruvate kinase M2 was optimal after Davidson’s fixation (Supplementary Fig. 2f; Table 2),

### Testing of methodological alterations

Overall, the results from the 49 antibodies tested on paraffin-embedded retinas revealed that NBF fixation produced superior immunolabelling to glyoxal fixation for 17, while glyoxal was better for just 3 of the antibodies (Table 2), with the remaining antibodies proving equally compatible with both fixatives. Fixation in Davidson’s solution produced superior immunolabelling to glyoxal fixation for 11 antibodies, while glyoxal was optimal for just 2 of the antibodies. As for the cryosection phase of the study, the data raise the question as to whether the protocol adopted for testing glyoxal was sub-optimal. Of significance, Dapson and colleagues^10^, while investigating the relationship between glyoxal fixation and immunohistochemistry, showed that HIAR using high pH Tris-HCl buffer was highly effective at unmasking epitopes in glyoxal fixed tissue. Therefore, we tested whether HIAR using Tris-HCl (pH 9) improved signal intensity in glyoxal-fixed retinas for antibodies that did not provide high signal-to-background labelling after HIAR using standard citrate buffer (pH 6). The results showed that for 12 of the 15 antibodies, HIAR using Tris-HCl, pH 9, improved signal intensity, although the increased specific labelling was almost invariably accompanied by more background staining (Supplementary Fig. 6; Supplementary Table 4). The mitochondrial proteins glutaminase and PDH showed the most striking differences, with Tris-HCl HIAR proving fully effective at unmasking these epitopes. Importantly, however, high pH Tris-HCl did not adequately address all of the limitations of glyoxal fixation. Immunolabelling for NFL, β_3_-tubulin, Hsp27, Hsp70, PNA, CD31 and cyclin D1 remained weaker than obtained after NBF fixation, yet each featured greater background staining. Importantly, the universal microglial marker, Iba1, still did not yield credible immunolabelling after HIAR with Tris-HCl, and the proliferation marker PCNA was extremely weak.

Lastly, we tested morphology and immunolabelling of paraffin-embedded eyes after fixation in glyoxal-v. Since glyoxal-v contains much higher concentrations of glyoxal and acetic acid than conventional glyoxal, we fixed some samples for 2 h and some for the standard duration of overnight. In terms of morphology, glyoxal-v retinas featured compact nuclear and plexiform layers with no artefactual damage; however, processing-induced retinal detachment did occur (Supplementary Fig. 7). There was no obvious difference between eyes fixed for 2 h or overnight. Regarding immunohistochemistry, we evaluated a panel of 20 antibodies in glyoxal-v eyes, in a side-by-side comparison with conventional glyoxal-fixed eyes, using whichever retrieval protocol worked best for glyoxal for each antibody. The data revealed that, for most antibodies, overnight fixation in glyoxal-v resulted in lower signal intensities as compared to conventional glyoxal, and also lower when compared to 2 h of fixation (Supplementary Fig. 8; Supplementary Table 5). The signal intensities of eyed fixed for 2 h in glyoxal-v were generally comparable to those fixed in conventional glyoxal (Supplementary Fig. 8; Supplementary Table 5). Of note, neither Iba1 antibody was reactive in glyoxal-v, mirroring results from the other phases of the study.

### Fixation regime considerations

For immunohistochemistry of the brain, it is routine for rodents to undergo transcardial perfusion with saline followed by fixative, prior to immersion fixation, as conducted by Konno et al.^13^ in their evaluation of glyoxal. This ensures rapid, consistent fixation of structures deep within the brain. This is not a prerequisite for retinal immunohistochemistry owing to the rapid penetration of fixative into the globe combined with the thin nature of the retina; thus, in this study, we employed immersion fixation. Nevertheless, to ensure that results are directly comparable with earlier published studies, we compared immunolabelling of wholemounts, cryosections and paraffin-embedded sections in eyes that had undergone immersion fixation alone versus those that had undergone transcardial perfusion with saline followed by glyoxal, prior to immersion fixation. We focussed only on selected antibodies that display sub-optimal immunolabelling with glyoxal fixation relative to formaldehyde. The data, presented in Supplementary Fig. 9, reveal, as expected, that the fixation regime had negligible influence on the patterns of immunolabelling. Signal intensities, for all antibodies tested, in all three formats (wholemounts, cryosections and paraffin-embedded sections) were similar irrespective of whether transcardial perfusion was employed prior to immersion fixation.

## Discussion

In the present study, we performed a systematic evaluation of the compatibility of glyoxal fixation with immunohistochemistry of the retina. Unlike the conclusions reached by authors of recent studies^10–13^, we found no compelling body of evidence that immunohistochemical signals are intensified when formaldehyde is replaced by glyoxal. In fact, for the 50 antibodies tested in this study, formaldehyde typically produced signal-to-background immunolabelling that was equivalent or superior to glyoxal. This was the case for retinal wholemounts, for cryosections prepared from eye cups, and for paraffin-embedded eyes. Testing of a different glyoxal formulation, as devised by Konno et al.^13^, which removes the ethanol component advocated by Richter and colleagues^11^ and substantially increases the concentrations of glyoxal and acetic acid, also failed to address the limitations of glyoxal as a fixative for the retina.

Immunolabelling of wholemounts is an essential methodology for studies evaluating retinal neuropathology and neurodegeneration in animal models of diseases^4,19,20^. The use of wholemounts facilitates identification of the entire population, or sub-populations, of different retinal cell types, including RGCs, amacrine cells, cone photoreceptors, micro- and macro-glia and vascular endothelial cells. Technically, immunolabelling of wholemounts is somewhat analogous to that of thick, free-floating vibratome tissue sections in that antibody penetration and epitope masking are both crucial limiting factors. As such, glyoxal should have presented significant advantages over formaldehyde. Glyoxal not only penetrates tissues better than formaldehyde, fixing tissues more rapidly and evenly, but, unlike formaldehyde, glyoxal does not cause slow, indiscriminate, progressive crosslinking of macromolecules, which can lead to conformational changes and disruption to epitopes^10,21^. Indeed, two recent publications that used free-floating brain slices both found that glyoxal fixation elicited signal intensification as compared to PFA^12,13^. Contrary to our expectations, glyoxal fixation was less suited to the wholemount methodology than PFA fixation. Physically, glyoxal-fixed retinas were too fragile to be consistently dissected as pristine wholemounts, particularly with the short fixation times that are commonly employed for their immunolabelling^22^. Moreover, the inclusion of ethanol in the glyoxal formulation devised by Richter et al.^11^ caused some patches of photoreceptor outer segments to adhere to the RPE rather than being dissected along with the retina, compromising any subsequent quantification of photoreceptor density. In terms of antigenicity, we observed no consistent increase when glyoxal fixation was used. While some antibodies produced higher signal intensities, a greater number displayed weaker signal-to-background patterns of labelling than after PFA fixation. The use of the alternative glyoxal formulation elicited signal intensities that were consistently inferior even to glyoxal. It needs to be acknowledged that there was little overlap in the panel of antibodies used herein and in published brain studies, nevertheless, the inconsistent performance of glyoxal in retinal wholemounts is surprising. It is plausible that the methodology – calibrated for immunolabelling PFA-fixed retinas – was sub-optimal for glyoxal. For example, Konno et al.^13^ noted that Triton X-100, which they incorporated into incubation and washing buffers, was essential for glyoxal-based immunohistochemistry in the brain. The employment of Triton X-100 is routine for immunolabelling retinal wholemounts^4^, however, the concentration used is often much higher than for brain vibratome slices. While future work may uncover an improved protocol for immunolabelling glyoxal-fixed wholemounts, the inherent fragility of such tissues will likely remain an issue.

Immunohistochemistry performed using NBF- or Davidson’s-fixed, paraffin-embedded retinas sections generally requires HIAR^5,23^. HIAR reverses the cumulative effects of fixation-induced cross-linking and antigen conformation modifications caused by the non-polar solvents used in tissue processing^24^. A persuasive logic for using glyoxal as a fixative for paraffin-embedded sections is that accessibility of antibody to target molecules should be greater due to the limited cross-linking that occurs, thereby largely eliminating the requirement for HIAR^9,13^. We did not find that this to be the case. Immunolabelling intensity was higher after HIAR for the majority of antibodies tested on glyoxal-fixed eyes. These findings match those of other studies that compared the effect of different fixatives on immunohistochemistry of breast cancer antigens in murine and human tissues^25,26^. Importantly, even with routine HIAR, a proportion of antibodies did not provide immunohistochemical labelling equivalent to NBF. For most of these antibodies, it was necessary to use HIAR with a high pH Tris-HCl buffer to produce satisfactory signal intensities, although the increased labelling was more often than not associated with added background staining. The requirement of certain antigens for HIAR comprising a high pH buffer has previously been reported for glyoxal-fixed tissue^10^. In general, HIAR using high pH buffers yields stronger immunohistochemical signals for most antibodies, irrespective of fixative^23^. Overall, glyoxal fixation, rather than eliminating the need for HIAR, actually increased complexity: the near-universal approach that suffices for NBF was not appropriate for glyoxal-fixed eyes. Some antigens needed no HIAR; some antigens required the use of an intermediate pH buffer; some antigens required a high pH buffer.

Two major weaknesses of NBF-fixed, paraffin-embedded eyes are suboptimal morphology and artefactual detachment of the retina. These deficiencies can be resolved by using Davidson’s solution, but at a cost of lower antigenicity for some molecules^5^. Glyoxal did not prove to be a “Goldilocks” fixative. Morphology of glyoxal-fixed retinas was better than in NBF-fixed eyes, but not as good as after Davidson’s fixation; moreover, some degree of retinal detachment occurred, which was presumably related to the lower concentration of ethanol in the glyoxal formulation compared with Davidson’s solution: 20% versus 33% respectively. Retinal detachment was even more pronounced when glyoxal-v was used, reflecting the lack of ethanol in this formulation. Irrespective of morphology, glyoxal-fixed retinas failed to address the reduced antigenicity that is associated with Davidson’s fixation. Of the panel of 49 antibodies tested, comparison of Davidson’s solution, glyoxal, and glyoxal-v revealed that a similar number of antibodies displayed weak immunoreactivity in each cohort. Overall, therefore, Davidson’s solution represents a much better option than glyoxal as an alternative fixative to NBF in pathological situations where optimal morphology and correct apposition of photoreceptors to the RPE are important.

The main goal of the present study was to compare the compatibilities of PFA and glyoxal with immunohistochemistry of the retina, as recent studies performed in the brain have shown that glyoxal fixation increases antigenicity^12,13^. Our results using paraffin-embedded eyes did not lead to this conclusion; however, paraffin sections undergo extensive chemical processing prior to immunolabelling, whereas fixation essentially represents the sole factor responsible for detecting an antigen in cryosections^27^. Thus, we directly compared immunolabelling in cryosections fixed in either PFA or glyoxal using an extensive panel of antibodies that have relevance to retinal research.

The first point of interest is that longer fixation in glyoxal reduced antigenicity. Of the 15 antibodies that displayed weak immunoreactivities after overnight fixation, 8 of these exhibited improved immunoreactivities when eyes were fixed for only 2 h. Whilst both recent brain publications used overnight glyoxal fixation^12,13^, this length of time appears to over-fix the retina. There is an inherent logic to this finding, as cryosections do not undergo HIAR, and PFA fixation of eyes to be used for cryosections is typically limited to 2 h due the globe being easily penetrated by fixatives Nevertheless, since glyoxal does not cause progressive crosslinking like PFA, the duration of fixation should theoretically have been less important. The disadvantage of only fixing for 2 h in glyoxal is that the resultant eye cups are soft and do not hold their shape, unlike PFA-fixed eye cups. Eyes that were fixed for 2 h in glyoxal-v were slightly more robust.

The second point of interest is that PFA was very widely compatible with the panel of antibodies tested. All 34 antibodies yielded specific immunolabelling in PFA-fixed retinas, the majority of which were rated in the high signal-to-background category. This is not particularly surprising. As noted by Stradleigh and Ishida in their authoritative review on fixation strategies for retinal immunohistochemistry^4^, formaldehyde-based fixatives have been so widely used in retinal immunohistochemistry that their usefulness is rarely questioned. This does not necessarily appear to translate to the brain. A case in point is the subset of antibodies directed against the vasculature and tight junction proteins. A recent brain publication indicated that glyoxal fixation is superior to PFA for labelling of these markers. Thomas et al.^12^ found that PFA-fixed brains displayed positive labelling for isolectin B4 and aquaporin-4, but not for CD31, laminin, ZO-1, occludin-1 or claudin-5, whereas all six markers could be successfully visualised in glyoxal-fixed brains. In our hands, antibodies directed against all six of these markers produced satisfactory and comparable signal intensities in PFA-fixed retinal cryosections.

The final point of interest is that glyoxal fixation did not increase antigenicity, rather it decreased antigenicity for a cohort of antibodies. Only calbindin and claudin-5 displayed unequivocally more intense signals after glyoxal fixation. The same antibodies that were problematic in paraffin eyes after glyoxal fixation were also challenging in cryosections, including the cytoskeletal proteins NFL and β_3_-tubulin, the neuronal markers parvalbumin and glutaminase, the cone photoreceptor marker PNA, various stress proteins, and, arguably most importantly, the microglial marker Iba1. Identification of microglia is vital in studies evaluating retinal neuropathology and neurodegeneration^28^, and Iba1 is a very widely used pan-marker of microglia. It is almost indispensable in paraffin sections, owing to the lack of reactivity of antibodies to CD11b. We were unable to produce adequate labelling for Iba1 after glyoxal fixation in cryosections, in paraffin sections, or in wholemounts. We tested two different antibodies to Iba1, each of which produced excellent labelling after formaldehyde fixation. The alternative glyoxal formulation, glyoxal-v, was completely incompatible with Iba1 antibodies, perhaps owing to the high concentration of acetic acid. Interestingly, Konno et al.^13^ achieved a positive signal for Iba1 in glyoxal-fixed brain sections, albeit accompanied by increased background staining.

In conclusion, the results of this study do not currently support the use of glyoxal fixation for immunohistochemistry of the rat retina, but with the caveat that improved formulations and protocols may address the limitations exposed herein.

## Electronic supplementary material

Below is the link to the electronic supplementary material.

Supplementary Material 1

## Data Availability

Any data will be made available upon request by contacting the corresponding author: glyn.chidlow@adelaide.edu.au.
