# Supplementary material for: Evaluation of Glyoxal fixation for immunohistochemistry of the retina
Source: Sci Rep. 2025 Jul 1;15:21274. doi: 10.1038/s41598-025-04226-7 (PMC12217606; doi:10.1038/s41598-025-04226-7)
Supplement: Supplementary file 1 — Supplementary Material 1 [file 41598_2025_4226_MOESM1_ESM.pdf]

**Supplementary Table 1A.** Antibodies used in the study

| Target | Host | *Clone/#Cat No. | Dilution | Source |
|---|---|---|---|---|
| aquaporin-4 | rabbit | #sc-20812 | 1:3000 | Santa-Cruz |
| Brn3a | goat | #sc-31984 | 1:3000 | Santa-Cruz |
| calbindin | mouse | *CB-955 | 1:1000 | Sigma |
| calretinin | mouse | #MAB1568 | 1:2500 | Merck-Millipore |
| cd11b | mouse | *OX-42 | 1:2000 | Bio-Rad |
| CD3 | rabbit | #A0452 | 1:3000 | Dako |
| CD31 | rat | #DIA-310 | 1:500; w1:100 | Dianova |
| CKMT1A | rabbit | #15346-1-AP | 1:5000 | Proteintech |
| claudin-5 | mouse | *4C3C2 | 1:1000 | Invitrogen |
| CNTF | goat | #AF 557-NA | 1:2000 | R&D Systems |
| cone arrestin | rabbit | #AB15282 | 1:10,000; w1:2500 | Merck-Millipore |
| COX IV | mouse | *20E8C12 | 1:3000 | Molecular Probes |
| CD68 | mouse | *ED1 | 1:500 | Bio-Rad |
| collagen VI | rabbit | #ab6588 | 1:1000 | Abcam |
| cyclin D1 | rabbit | #ab21699 | 1:10 | Abcam (prediluted) |
| FGF-2 | mouse | *bFM-2 | 1:500 | Merck-Millipore |
| GFAP | rabbit | #Z0 334 | 1:40,000; w1:4000 | Dako |
| glutaminase | rabbit | #HPA036223 | 1:1000 | Sigma |
| glutamine synthetase | mouse | #610517 | 1:1000 | BD transduction |
| Hsp27 | rabbit | #SPA-801 | 1:2500 | Enzo Life Sciences |
| Hsp70 | mouse | *C92F3A-5 | 1:500 | Enzo Life Sciences |
| Iba1 | goat | #NB100-1028 | 1:50,000, w1:5000 | Novus Biologicals |
| Iba1 | rabbit | #019-19741 | 1:40,000, w1:4000 | WAKO |
| IL-1β | goat | #AF 501 NA | 1:3000 | R&D Systems |
| laminin | rabbit | #AT 2404 | 1:3000 | E Y Labs |
| MAP2 | mouse | #MAB3418 | 1:1000 | Merck-Millipore |
| melanopsin | rabbit | #PA1-780 | 1:2000; w1:400 | Invitrogen |
| MCT1 | chicken | #AB1286 | 1:5000 | Merck-Millipore |

| | | | | |
|---|---|---|---|---|
| M/L-opsin | rabbit | #AB5405 | 1:10,000 | Merck-Millipore |
| Myeloperoxidase | rabbit | #A 0398 | 1:100,000 | Dako |
| nestin | mouse | *Rat 401 | 1:1000 | BD transduction |
| np-NFH | mouse | *SMI-32 | 1:10,000 | Covance |
| NFL | mouse | #MAB1615 | 1:2000 | Merck-Millipore |
| occludin-1 | mouse | #33-1500 | 1:1000 | Invitrogen |
| PCNA | mouse | *PC10 | 1:10,000 | Dako |
| PKCα | rabbit | #2056 | 1:1000 | CST |
| PKM2 | rabbit | #4053 | 1:2500 | CST |
| RBPMS | rabbit | #NBP2-20112 | 1:3000; [w]1:750 | Novus Biologicals |
| RPE65 | mouse | *8B11 | 1:3000 | Santa-Cruz |
| rhodopsin | mouse | *RET-P1 | 1:1000 | Santa-Cruz |
| S100 | rabbit | #Z0311 | 1:50,000 | Dako |
| synaptophysin | mouse | *SY38 | 1:5000 | Dako |
| syntaxin-1 | mouse | #S0664 | 1:3000 | Sigma |
| χ-synuclein | mouse | *CPTC-SNCG-1 | 1:1000; [w]1:200 | DSHB |
| $\beta_3$-tubulin | rabbit | #D65A4 | 1:1000 | CST |
| vGLUT | mouse | #sc-377425 | 1:1000 | Santa-Cruz |
| vimentin | mouse | *V9 | 1:1000 | Dako |
| ZO-1 | rabbit | #40-2200 | 1:2000 | Invitrogen |

[w]dilution used for 2-step wholemount immunostaining; CST, Cell Signaling Technology; DSHB, Developmental Studies Hybridoma Bank

**Supplementary Table 1B.** Lectins used in the study

| Target | Host | *Clone/#Cat No. | Dilution | Source |
|---|---|---|---|---|
| isolectin B4 | biotinylated | #L2140 | 1:750 | Sigma |
| Peanut agglutinin | biotinylated | #B-1075 | 1:10,000 | Vector Laboratories |

**Supplementary Table 2.** Summary of compatibility of miscellaneous antibodies with glyoxal-variant (glyoxal-v) fixed, retinal wholemounts

| Target | Labelling intensity | | Target | Labelling intensity | |
|---|---|---|---|---|---|
| | glyoxal-v (24h fix) | Glyoxal-v (2h fix) | | glyoxal-v (24h fix) | Glyoxal-v (2h fix) |
| cd11b | - | - | PNA | -/+ | -/+ |
| cone arrestin | +++ | +++ | RBPMS | ++ | ++ |
| iba1 | - | - | χ-synuclein | + | + |

Grading scheme: - = minimal specific labelling; + = weak specific labelling; ++ = modest specific labelling; +++ = intense specific labelling.

**Supplementary Table 3.** Comparison of different glyoxal fixation protocols on antibody labelling intensity in retinal cryosections

| Target | Labelling intensity: glyoxal (overnight fix) | Labelling intensity: glyoxal (2h fix) | Labelling intensity: glyoxal-v (2h fix) | Target | Labelling intensity: glyoxal (overnight fix) | Labelling intensity: glyoxal (2h fix) | Labelling intensity: glyoxal-v (2h fix) |
|---|---|---|---|---|---|---|---|
| CD11b | +++ | ++ | -/+ | Iba1 (WAKO) | + | + | -/+ |
| CD31 | + | ++ | +++ | MCT1 | ++ | ++/+++ | ++ |
| cone arrestin | ++ | ++ | ++ | nestin | + | ++ | ++ |
| FGF-2 | - | - | - | NFL | +/++ | ++ | ++ |
| glutaminase | - | - | - | parvalbumin | +/++ | + | +/++ |
| Hsp27 | + | + | - | PNA | + | ++ | + |
| Hsp70 | + | +++ | ++/+++ | $\beta_3$-tubulin | + | ++ | +/++ |
| Iba1 (Novus) | + | +/++ | - | | | | |

Grading scheme: - = minimal specific labelling; + = weak specific labelling; ++ = modest specific labelling; +++ = intense specific labelling.

**Supplementary Table 4.** Comparison of heat-induced antigen retrieval protocols on antibody signal intensity in glyoxal-fixed, paraffin-embedded retinas

| Target | Labelling intensity | | Target | Labelling intensity | |
|---|---|---|---|---|---|
| | Citrate, pH 6 | Tris-HCl, pH 9 | | Citrate, pH 6 | Tris-HCl, pH 9 |
| CD31 | ++ | ++ | nestin | ++ | +++ |
| Cone arrestin | ++/+++ | +++ | NF-L | ++ | +++ |
| Cyclin D1 | + | ++ | parvalbumin | + | +/++ |
| glutaminase | -/+ | +++ | PCNA | - | -/+ |
| Hsp27 | + | +/++ | PDH | + | ++/+++ |
| Hsp70 | + | +/++ | PNA | +/++ | +/++ |
| Iba1 (Novus) | -/+ | + | $\beta_3$-tubulin | + | +/++ |
| Iba1 (WAKO) | -/+ | -/+ | | | |

Grading scheme: - = minimal specific labelling; + = weak specific labelling; ++ = modest specific labelling; +++ = intense specific labelling.

**Supplementary Table 5.** Comparison of different glyoxal fixation protocols on antibody labelling intensity in paraffin-embedded retinas

| | Labelling intensity | | | | Labelling intensity | | |
|---|---|---|---|---|---|---|---|
| Target | glyoxal (24h fix) | Glyoxal-v (24h fix) | glyoxal-v (2h fix) | Target | glyoxal (24h fix) | Glyoxal-v (24h fix) | glyoxal-v (2h fix) |
| aquaporin-4 | ++/+++ | ++/+++ | +++ | laminin | +++ | + | +++ |
| calbindin | +++ | ++ | +++ | melanopsin | +++ | ++ | +++ |
| CD31 | ++/+++ | + | ++/+++ | NFL | +++ | ++ | +++ |
| Claudin-5 | ++ | + | ++ | parvalbumin | + | + | +/++ |
| cone arrestin | +++ | ++ | ++/+++ | PKC | +++ | +++ | +++ |
| glutaminase | +++ | ++/+++ | ++/+++ | PNA | +/++ | + | ++ |
| Hsp27 | +/++ | -/+ | -/+ | RBPMS | +++ | +++ | +++ |
| Hsp70 | +/++ | -/+ | + | rhodopsin | +++ | ++/+++ | ++/+++ |
| Iba1 (Novus) | + | - | - | S100 | +++ | +++ | ++/+++ |
| Iba1 (WAKO) | + | - | - | $\beta_3$-tubulin | +/++ | + | +/++ |

Grading scheme: - = minimal specific labelling; + = weak specific labelling; ++ = modest specific labelling; +++ = intense specific labelling.

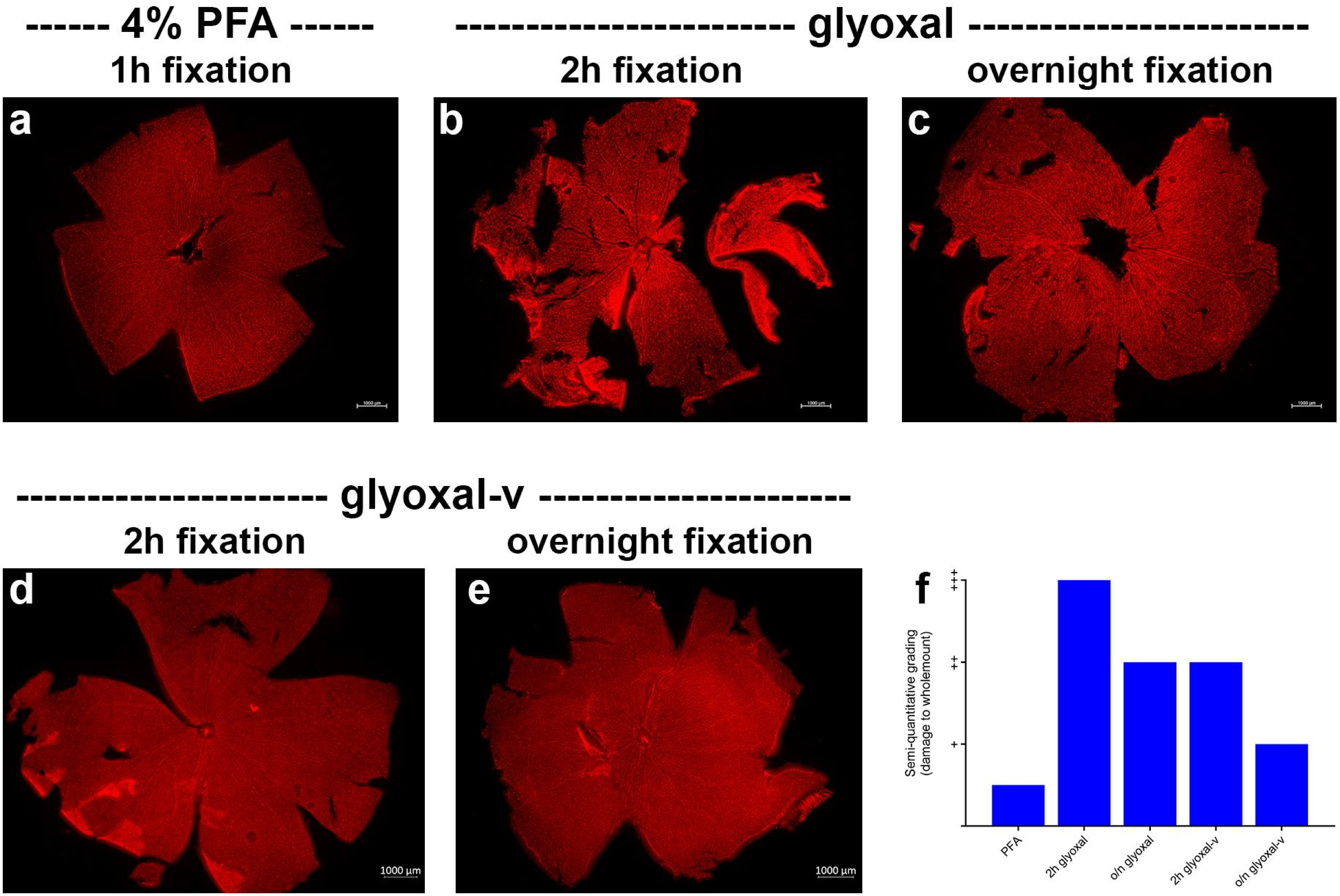


**Supplementary Fig. 1.** Representative images of PFA- and glyoxal-fixed retinal wholemount morphology, as delineated by fluorescent immunohistochemistry for RBPMS. (a) Retina fixed for 1 hour (h) in PFA displays excellent structural integrity. (b) Retina fixed for 2h in glyoxal features artefactual tears and folds. (c) Retina fixed overnight in glyoxal shows greater structural preservation than after 2h fixation, but still has artefactual tears and holes. (d, e) Retinas fixed for 2h and overnight in glyoxal-v feature better morphology than after the equivalent times in glyoxal. (f) Semi-quantitative grading scheme of structural integrity of retinal wholemounts after different fixations.

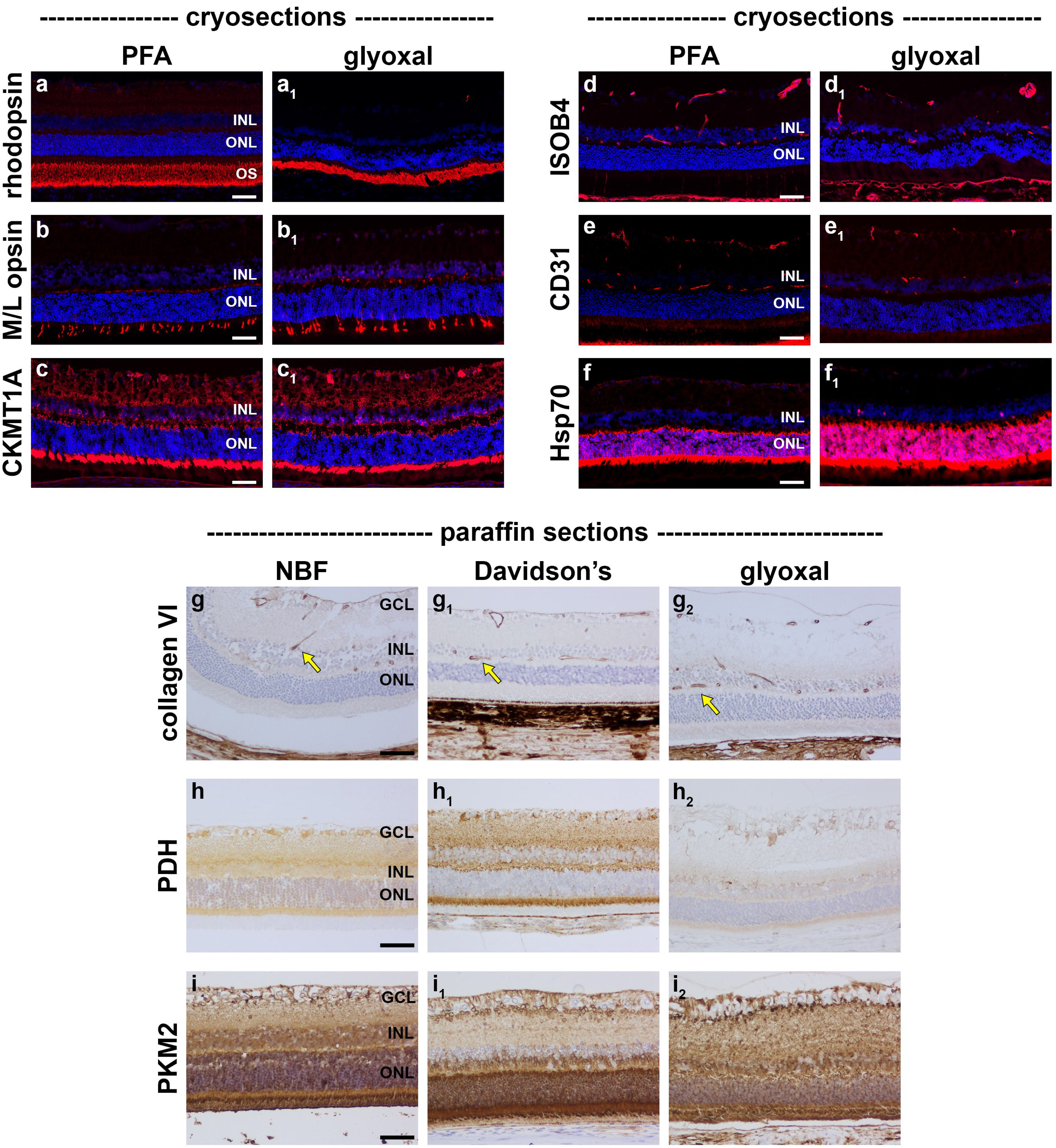


**Supplementary Fig. 2.** Representative images of various antibodies in PFA- and glyoxal-fixed retinal cryosections (a-c), or, in NBF-, Davidson's- and glyoxal-fixed, paraffin-embedded sections of retina (d-f), as delineated by fluorescent or colorimetric immunohistochemistry. (a, a1, a2) Rod photoreceptor segments labelled by rhodopsin. (b, b1, b2) Cone photoreceptor segments labelled by M/L opsin. (c, c1, c2) Mitochondria labelled by ubiquitous mitochondrial creatine kinase subunit 1A (CKMT1A).(d, d1, d2) Blood vessels labelled by isolectin B4 (ISOB4). (e, e1, e2) Blood vessels labelled by CD31. (f, f1, f2) Photoreceptors labelled by Hsp70. (g, g1, g2) The inner limiting membrane and blood vessels (arrows) labelled by collagen VI. (h, h1, h2) Mitochondria labelled by pyruvate dehydrogenase (PDH). PDH immunolabelling was excellent with Davidson's fixation, displayed modest signal-to-background after NBF fixation and was only very weakly reactive after glyoxal fixation. (i, i1, i2) Labelling for the glycolytic pyruvate kinase isoenzyme PKM2. PKM2 immunolabelling was excellent with Davidson's fixation, but displayed modest signal-to-background after NBF and glyoxal fixation. Scale bars: a-c = 50µm; d-f = 60µm. GCL, ganglion cell layer; INL, inner nuclear layer; ONL, outer nuclear layer; OS, outer segments.

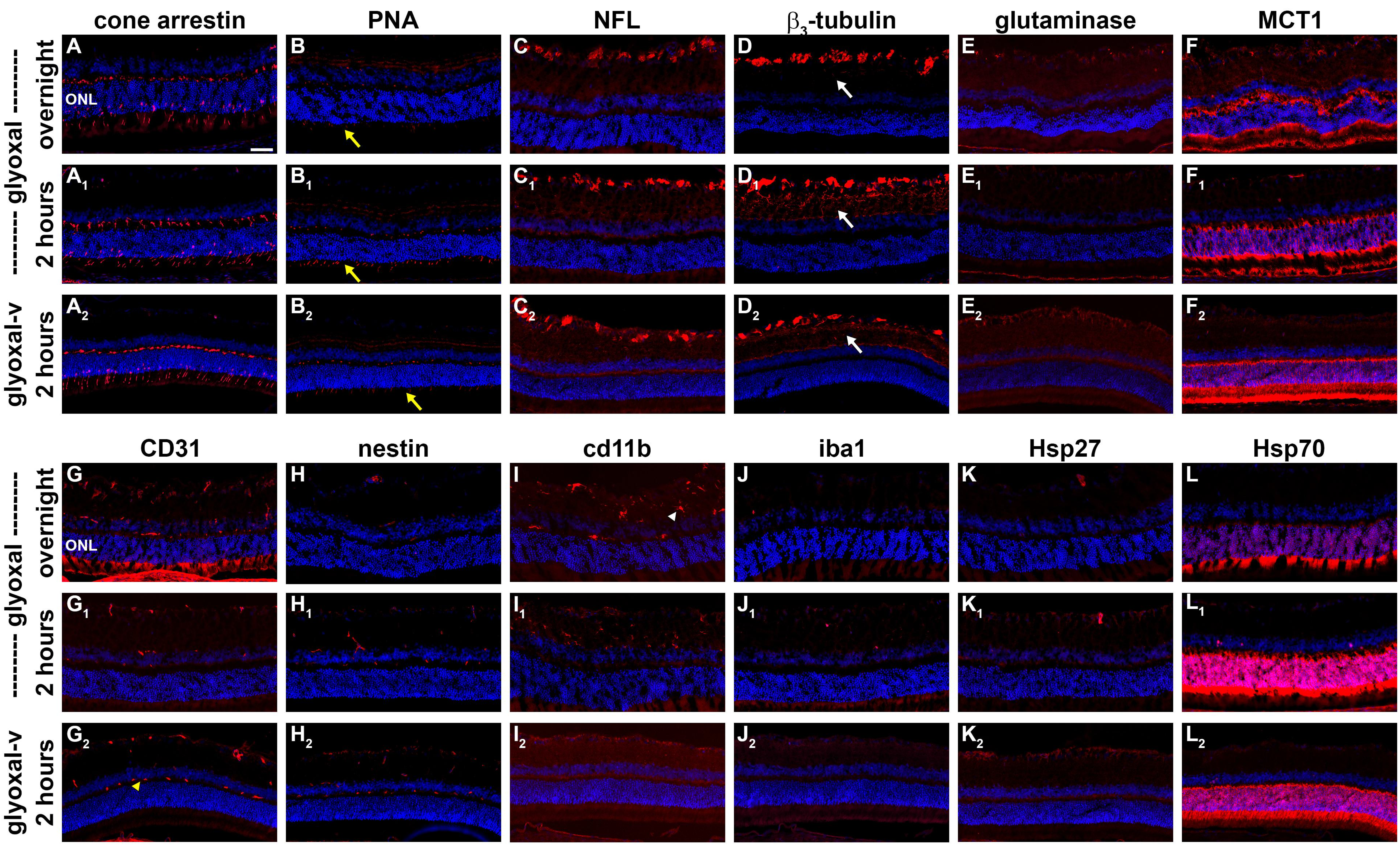


**Supplementary Fig. 3.** Representative images of various antibodies in retinal cryosections from eyes fixed either overnight in glyoxal, or for 2 hours in glyoxal, or for 2 hours in glyoxal variant (glyoxal-v), as delineated by fluorescent immunohistochemistry. (A, A1, A2) Cone photoreceptors labelled by cone arrestin. (B, B1, B2) Cone photoreceptor segments (yellow arrows) labelled by peanut agglutinin (PNA). (C, C1, C2) RGCs labelled by NFL. (D, D1, D2) RGC axon bundles and dendrites (white arrows) labelled by $\beta_3$-tubulin. (E, E1, E2) Neurons labelled by glutaminase. (F, F1, F2) Blood vessels, photoreceptors and the RPE labelled by the monocarboxylate transporter MCT1. (G, G1, G2) Blood vessels labelled by CD31. Signal-to-background was optimal with glyoxal-v (yellow arrowhead). (H, H1, H2) Blood vessels labelled by nestin. Antigenicity was improved by shorter duration of fixation. (I, I1, I2) Microglia labelled by cd11b. Cd11b immunolabelling was excellent with overnight glyoxal fixation (white arrowhead), modest after 2h glyoxal fixation and non-reactive with glyoxal-v. (J, J1, J2) Microglia labelled by Iba1. Iba1 immunolabelling was faintly discernible after glyoxal fixation, but not with glyoxal-v. (K, K1, K2) Hsp27 labelling. Neither astrocytes nor blood vessels are readily visible with any of the fixative protocols. (L, L1, l2) Photoreceptor somata and segments labelled by Hsp70. Immunolabelling was weak after overnight glyoxal fixation, but excellent with shorter duration of fixation. ONL, outer nuclear layer. Scale bar: 50µm.

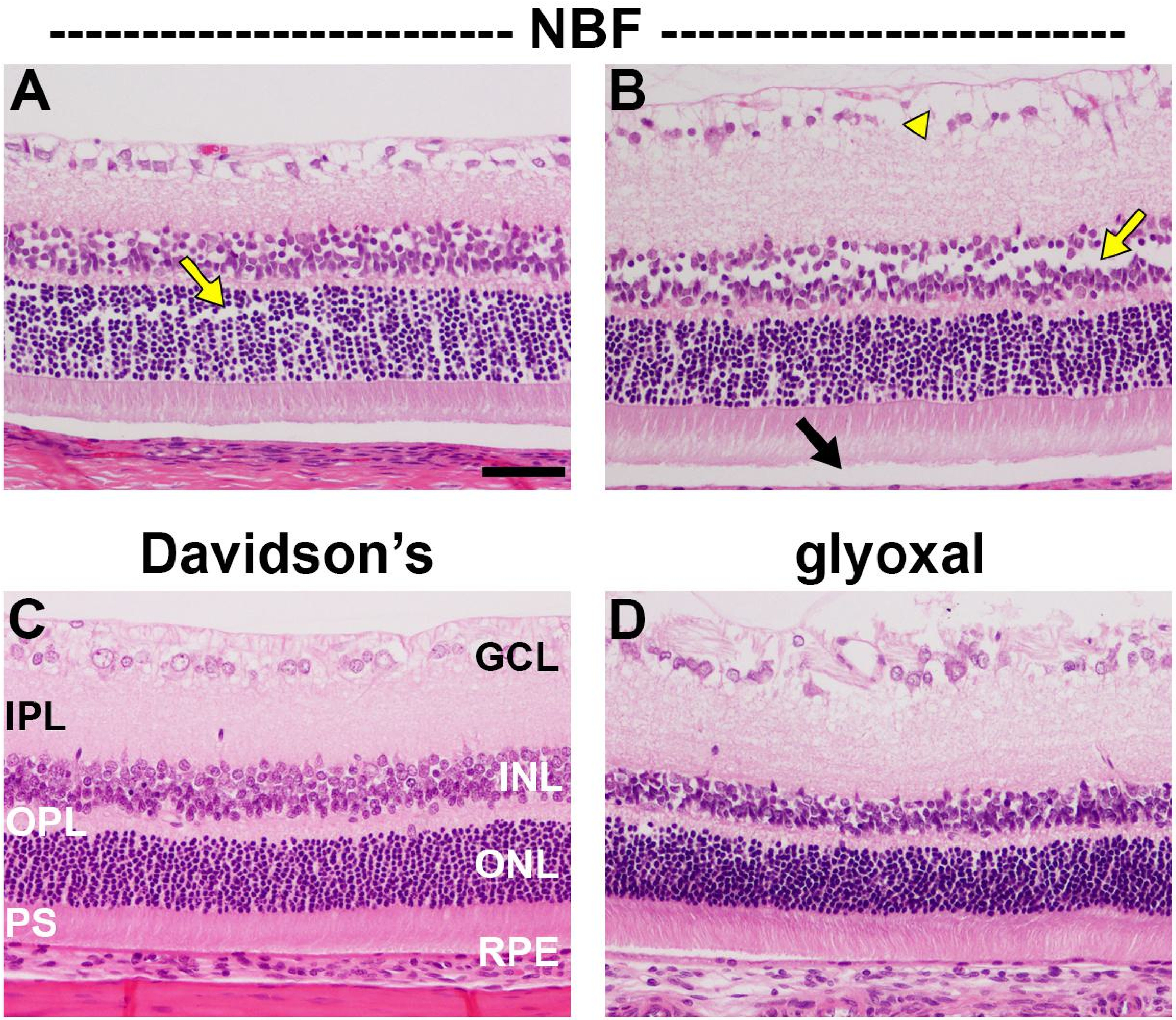


**Supplementary Fig. 4.** Representative images of retinal histology after NBF (A, B), Davidson's (C) and glyoxal (D) fixation, as delineated by H&E staining in paraffin-embedded eyes. Artefactual separation of the nuclear layers (yellow arrows and arrowheads) and detachment of the retina (black arrow) is typically observed in NBF-fixed eyes. Scale bar: 60 µm. GCL, ganglion cell layer; IPL, inner plexiform layer; INL, inner nuclear layer; OPL, outer plexiform layer; ONL, outer nuclear layer; PS, photoreceptor segments; RPE, retinal pigment epithelium.

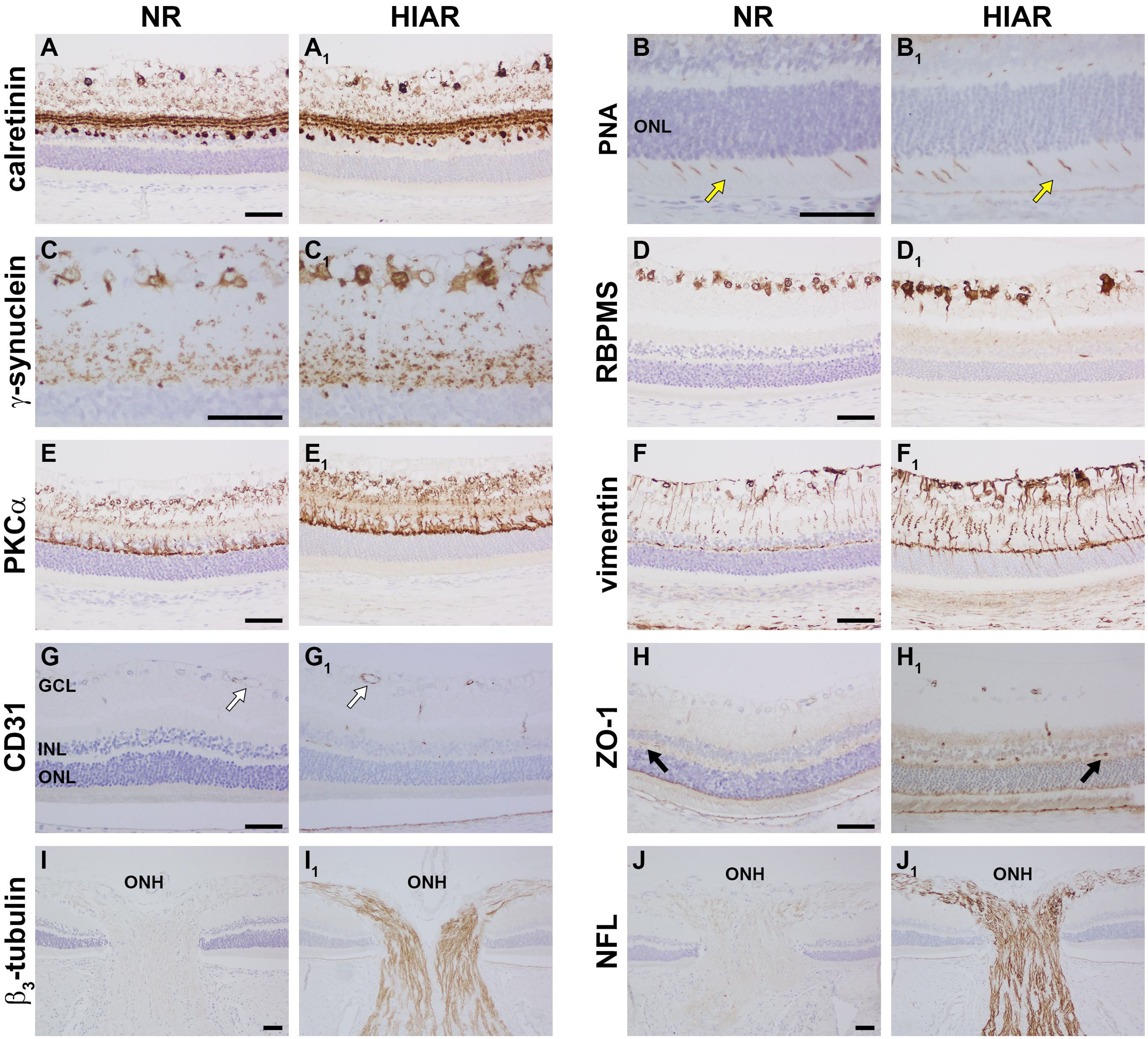


**Supplementary Fig. 5**. Representative images of various antibodies in glyoxal-fixed, paraffin-embedded sections of retina that underwent either no antigen retrieval (NR) or heat-induced antigen retrieval (HIAR), as delineated by colorimetric immunohistochemistry. (A, A1) Amacrine cells and RGCs labelled by calretinin with 3 layers of terminals visible. HIAR does not noticeably enhance signal intensity (B, B1) Cone photoreceptor segments labelled by peanut agglutinin (PNA; yellow arrows) HIAR does not noticeably enhance signal intensity. (C, C1) RGC somas and axons labelled by g-synuclein. HIAR marginally enhances signal intensity. (D, D1) RGC somas labelled by RBPMS. HIAR marginally enhances signal intensity. (E, E1) Rod bipolar cells and their processes terminating in the inner and outer plexiform layers labelled by PKCα. HIAR enhances signal intensity. (F, F1) Astrocytes and Müller cell processes labelled by vimentin. HIAR enhances signal intensity. (G, G1) Blood vessels labelled by CD31. HIAR enhances signal intensity (white arrows) but increases background labelling. (H, H1) Tight junctions forming the blood-retinal barrier labelled by ZO-1. HIAR enhances signal intensity (black arrows) but increases background labelling. (I, I1) RGC axon bundles labelled by β3-tubulin. HIAR greatly enhances signal intensity. (J, J1) RGC axon bundles labelled by NFL. HIAR greatly enhances signal intensity. Scale bar: 60μm. GCL, ganglion cell layer; INL, inner nuclear layer; ONH, optic nerve head; ONL, outer nuclear layer

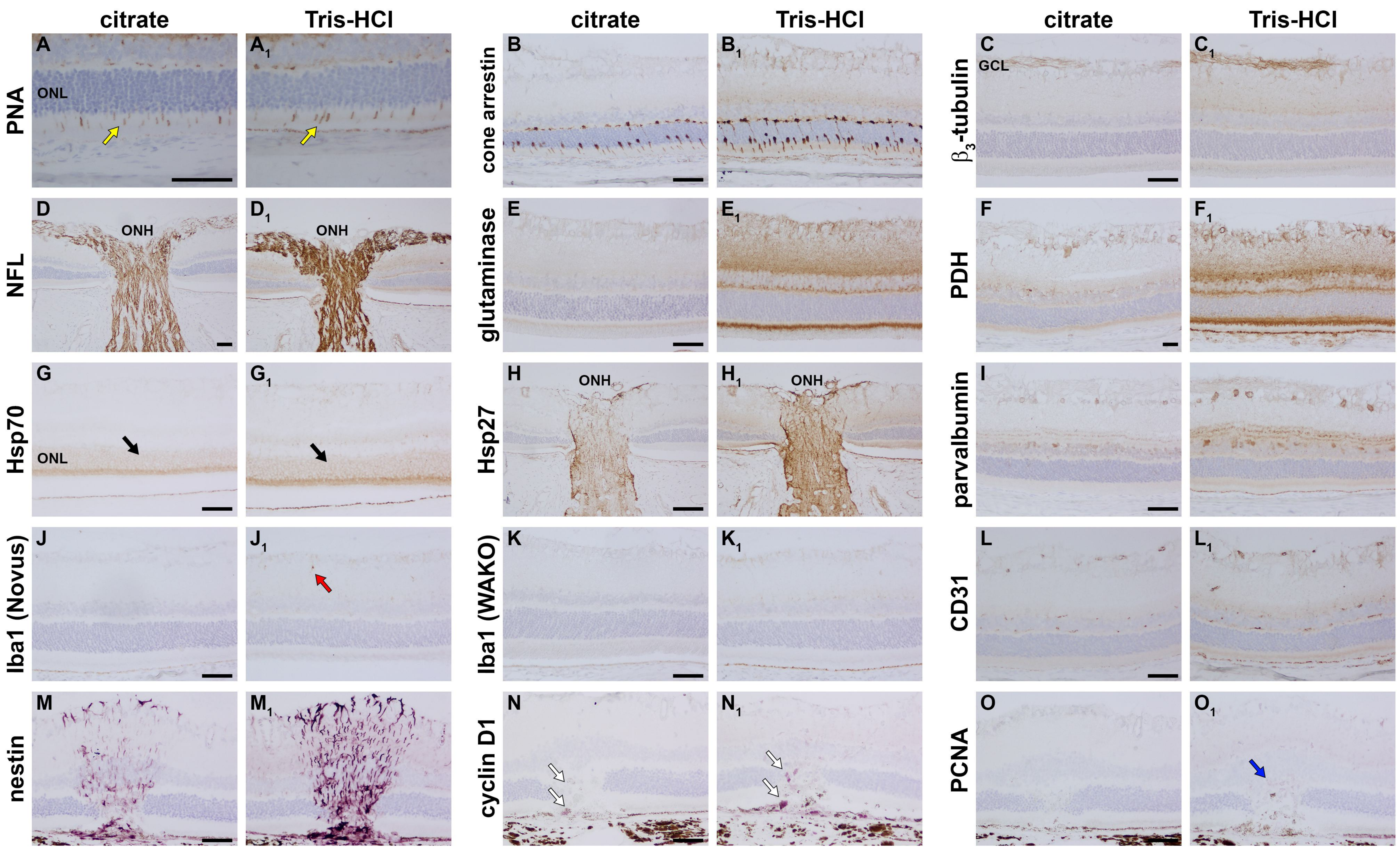


**Supplementary Fig. 6.** Representative images of various antibodies in glyoxal-fixed, paraffin-embedded sections of retina that underwent heat-induced antigen retrieval (HIAR) using either 10 mM citrate buffer (pH 6) or 100 mM Tris-HCl (pH 9), as delineated by colorimetric immunohistochemistry. (A, A1) Cone photoreceptor segments labelled by peanut agglutinin (PNA; y ellow arrows). HIAR does not noticeably enhance signal intensity. (B, B1) Cone photoreceptor somata together with their axon terminals and segments labelled by cone arrestin. (C, C1) RGC somata, dendrites and axon bundles labelled by $\beta_3$-tubulin. (D, D1) RGC axons at the optic nerve head (ONH) labelled by NFL. (E, E1) Glutamatergic neurons labelled by glutaminase. (F, F1) Expression of the mitochondrial enzyme pyruvate dehydrogenase (PDH). (G, G1,) Photoreceptor somata and segments (black arrows) labelled by the inducible heat shock protein Hsp70. (H, H1) Astrocytes at the ONH labelled by the inducible heat shock protein Hsp27. (I, I1) Inner retinal neurons labelled by parvalbumin. (J, J1) Microglia labelled by Iba1 (Novus antibody; red arrows). (K, K1) Microglia labelled by Iba1 (WAKO antibody). (L, L1) Blood vessels labelled by CD31. ¬(M, M1) Nestin expression at the site of laser lesion. (N, N1) Cyclin D1-positive cells at the site of laser lesion (white arrows). (O, O1) PCNA-positive cells at the site of laser lesion (blue arrow). In all cases, signal intensity was higher after performing HIAR with Tris-HCl. Scale bars: 60 µm. GCL, ganglion cell layer; outer nuclear layer.

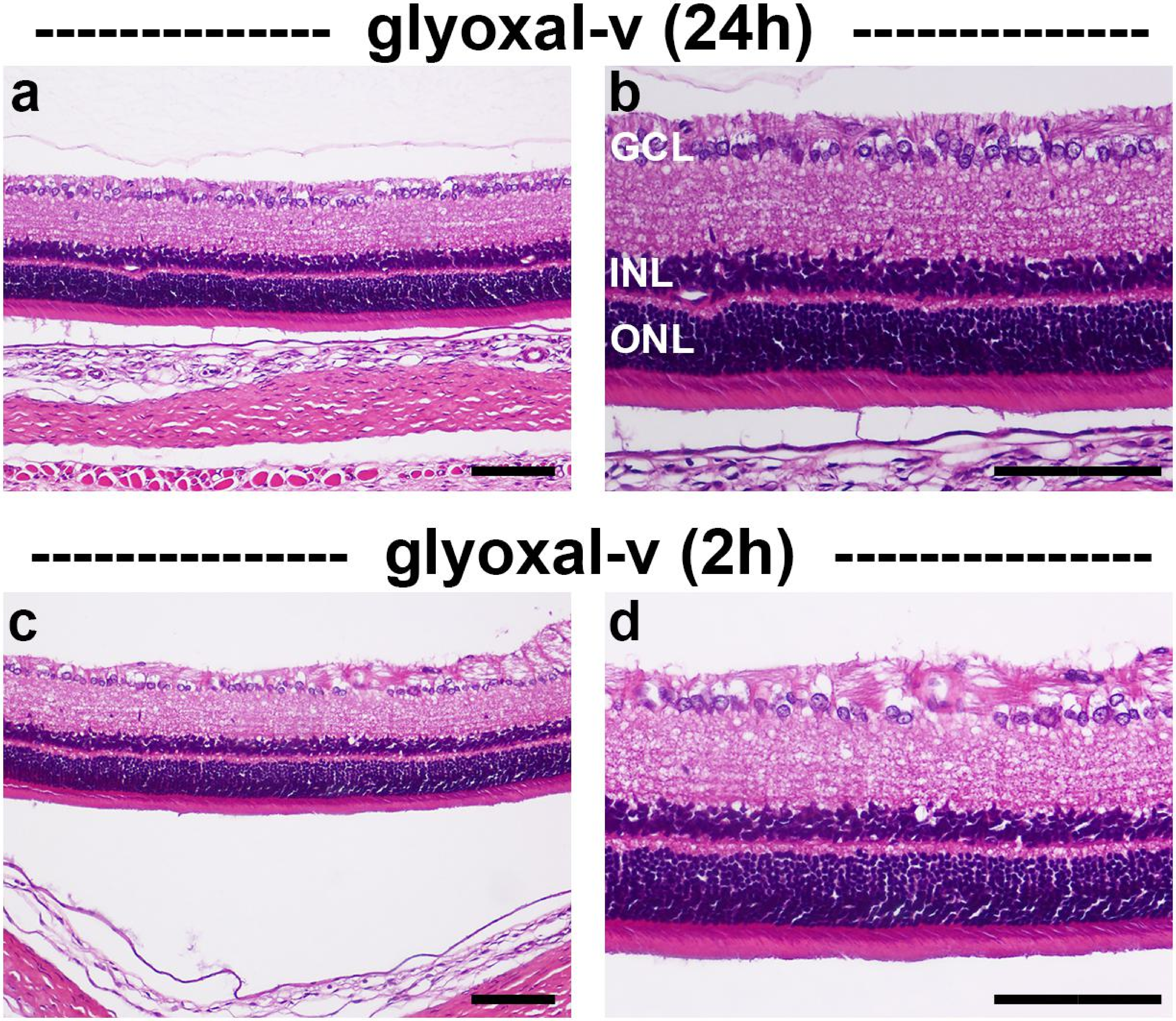


**Supplementary Fig. 7**. Representative images of retinal histology after fixation in glyoxal variation (glyoxal-v) for 24 hours (A, B) or 2 hours (C, D), as delineated by H&E staining in paraffin-embedded eyes.

In each case, the retina features compact \nuclear layers, but artefactual detachment of the retina. There is minimal difference in morphology between 2 and 24 hour fixation. Scale bars: 120 µm. GCL, ganglion cell layer; INL, inner nuclear layer; ONL, outer nuclear layer.

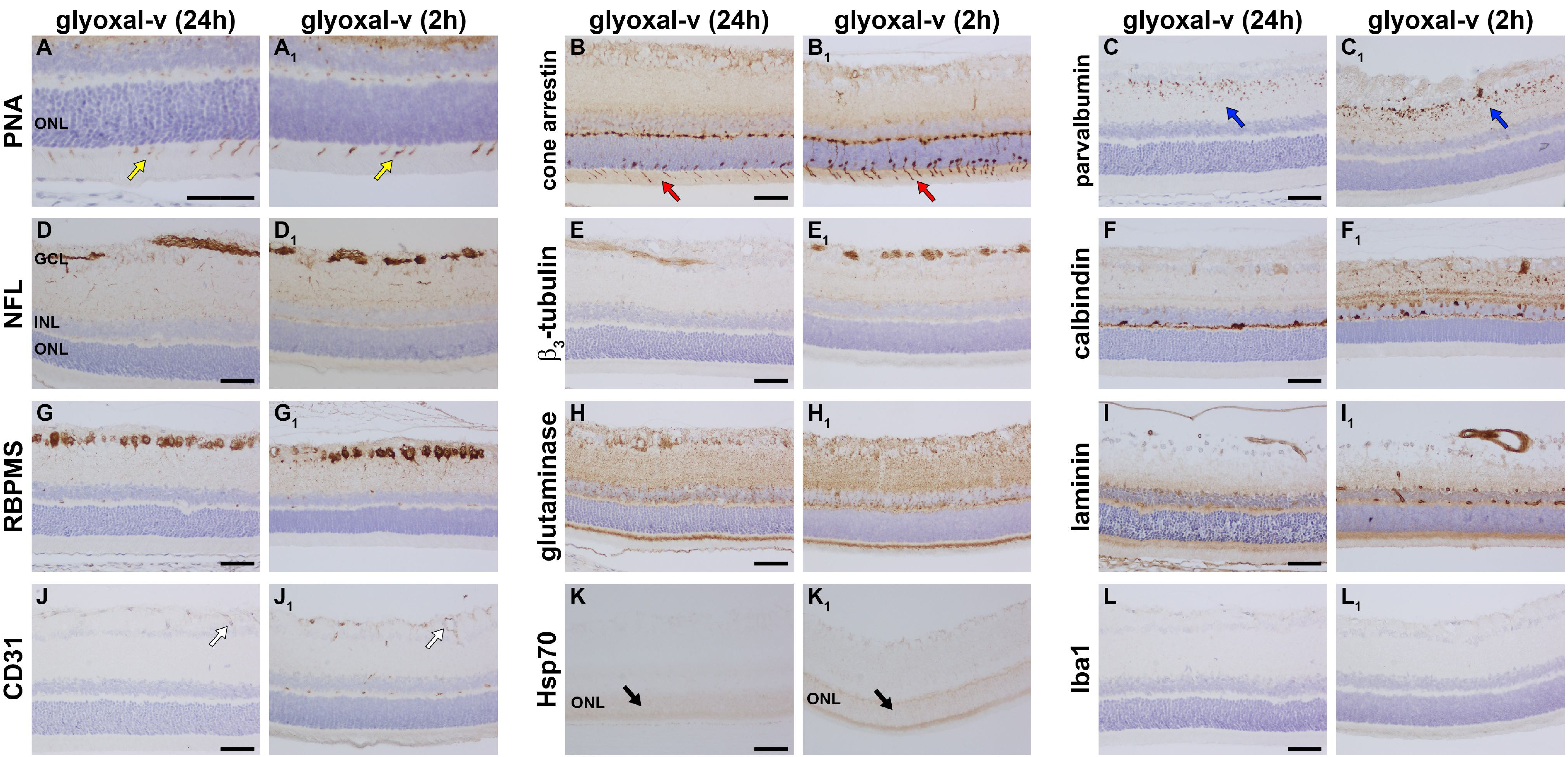


**Supplementary Fig. 8**. Representative images of various antibodies in paraffin-embedded sections from eyes fixed for 24 hours in glyoxal variant (glyoxal-v), or for 2 hours in glyoxal-v, as delineated by colorimetric immunohistochemistry. For all antibodies reactive in glyoxal-v, signal intensity was higher after the shorter fixation time. (A, A1) Cone photoreceptor segments labelled by peanut agglutinin (PNA; yellow arrows). (B, B1) Cone photoreceptor somata together with their axon terminals and segments (red arrows. (C, C1) Amacrine cells and processes labelled by parvalbumin (blue arrows). (D, D1) RGC somata, dendrites and axon bundles labelled by NFL. (E, E1) RGC somata, dendrites and axon bundles labelled by $\beta_3$-tubulin. (F, F1) Inner retinal neurons and their processes labelled by calbindin. (G, G1) RGCs labelled by RBPMS. (H, H1) Glutamatergic neurons labelled with the enzyme glutaminase. (I, I1) Blood vessels labelled by laminin. (J, J1) Blood vessels labelled by CD31 (white arrows). (K, K1) Photoreceptor somata and segments (black arrows) labelled by the inducible heat shock protein Hsp70. (L, L1) Microglia labelled by Iba1. Antibodies to Iba1 were non-reactive irrespective of fixation time. Scale bars: 60 μm. GCL, ganglion cell layer; INL, inner nuclear layer; ONL, outer nuclear layer.

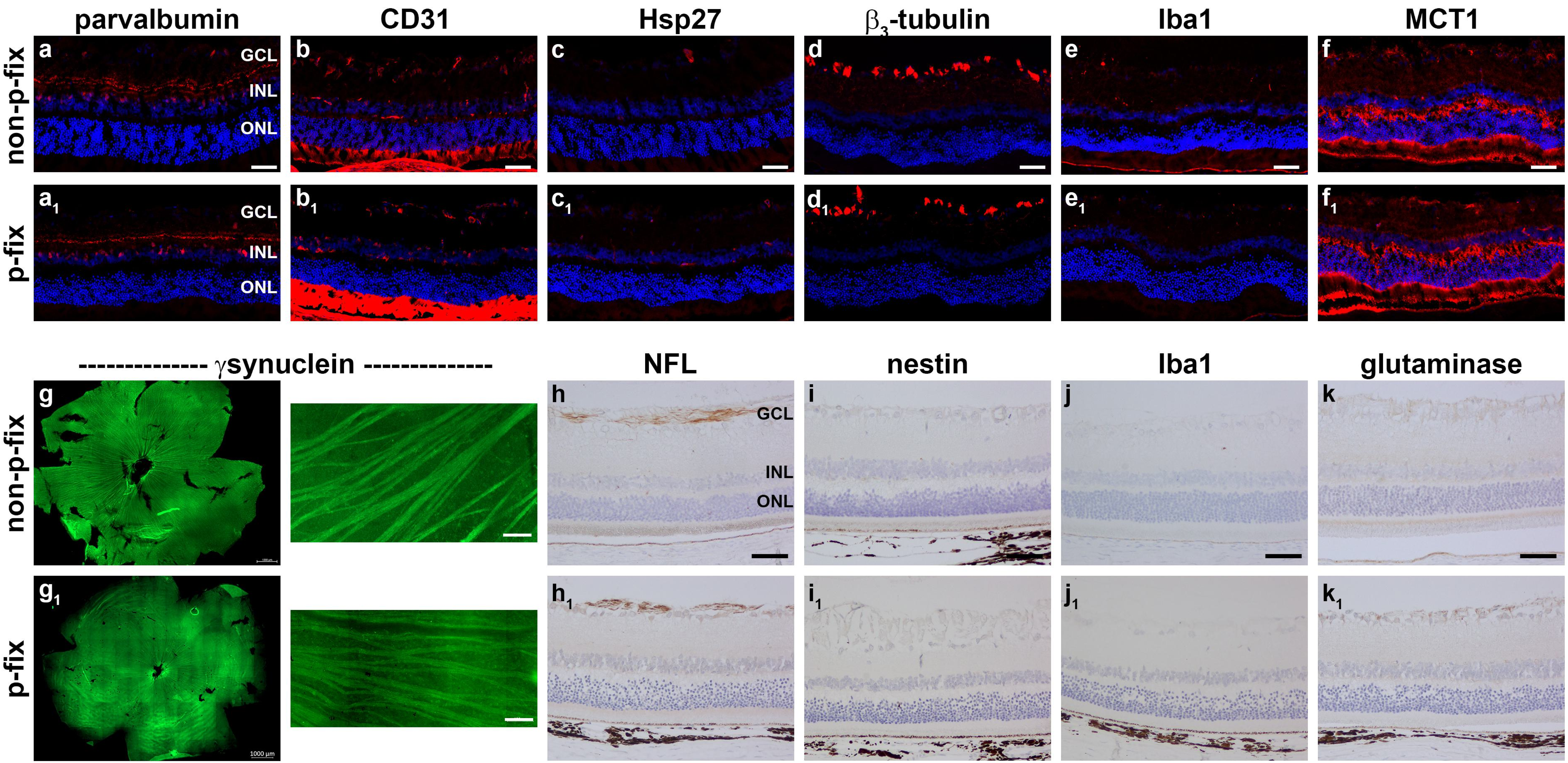


**Supplementary Fig. 9.** Representative images of various antibodies in glyoxal-fixed, cryosections (a-f), wholemounts (g) and paraffin-embedded sections (h-k) of retina that underwent either immersion fixation, or, transcardial perfusion with saline followed by glyoxal and were subsequently immersion fixed. (a, a1) Parvalbumin labelling. (b, b1) CD31 labelling. (c, c1) Hsp27 labelling. (d, d1) $\beta_3$-Tubulin labelling. (e, e1) Iba1 labelling. (f, f1) MCT1 labelling. (g, g1) Wholemounts labelled by g-synuclein. (h, h1) NFL labelling. (i, i1) Nestin labelling. (j, j1) Iba1 labelling. (k, k1) glutaminase labelling. Scale bars: a-f = 50µm; g = 1000µm **(**overview**),** and 100µm (adjacent zoomed image); h-k = 60µm; GCL, ganglion cell layer; INL, inner nuclear layer; ONH, optic nerve head; ONL, outer nuclear layer.
